# The Effects of Anthropogenic Stressors on Above- and Belowground Phytochemical Diversity of the Wetland Grass, *Phragmites australis*

**DOI:** 10.3390/plants13223133

**Published:** 2024-11-07

**Authors:** Andrea E. Glassmire, Ana L. Salgado, Rodrigo Diaz, Joseph Johnston, Laura A. Meyerson, Joshua S. Snook, James T. Cronin

**Affiliations:** 1 Department of Biological Sciences, Louisiana State University, Baton Rouge, LA 70803, USA; amald@lsu.edu (A.L.S.); 3johnstonjoe@gmail.com (J.J.); jcronin@lsu.edu (J.T.C.); 2 Department of Entomology, Louisiana State University, Baton Rouge, LA 70803, USA; rdiaz@agcenter.lsu.edu (R.D.); jsnook@agcenter.lsu.edu (J.S.S.); 3 Department of Natural Resources Science, University of Rhode Island, Kingston, RI 02881, USA; lameyerson@uri.edu

**Keywords:** phytochemical diversity, metabolomics, climate change, salt marsh, Roseau cane scale, non-native species, intraspecific, machine learning

## Abstract

Coastal wetlands face threats from climate change-induced flooding and biological invasions. Plants respond to these stressors through changes in their phytochemical metabolome, but it is unclear whether stressors affecting one tissue compartment (e.g., leaves) create vulnerabilities in others (e.g., roots) or elicit similar responses across tissues. Additionally, responses to multiple simultaneous stressors remain poorly understood due to the focus on individual metabolites in past studies. This study aims to elucidate how the phytochemical metabolome of three *Phragmites australis* (Cav.) lineages, common in the Mississippi River Delta, responds to flooding and infestation by the non-native scale insect *Nipponaclerda biwakoensis* (Kuwana). Among these lineages, one is non-native and poses a threat to North American wetlands. Results indicate that metabolomic responses are highly specific, varying with lineage, tissue type, stressor type, and the presence of multiple stressors. Notably, the non-native lineage displayed high chemical evenness, while the other two showed stressor-dependent responses. The 10 most informative features identified by a machine learning model showed less than 1% overlap with known metabolites linked to water and herbivory stress, underscoring gaps in our understanding of plant responses to environmental stressors. Our metabolomic approach offers a valuable tool for identifying candidate plant genotypes for wetland restoration.

## 1. Introduction

Natural plant communities are under threat due to myriad factors that are attributed to global climate change and the encroachment of non-native species. Higher temperatures, rising sea levels and storm surge caused by hurricanes have contributed to more intense flooding [1]. The introduction of non-native species has also increased worldwide, with more than one-third of all introductions occurring between 1970 and 2014 [2]. Coastal ecosystems are particularly under threat from flooding and non-native species [3,4], and, like other natural communities, these anthropogenic threats can cascade from plants to the entire wetland community [5,6,7]. An important yet understudied avenue through which these cascades can occur is via chemical changes in the plant community. Shifts in plant chemical profiles may influence interactions with herbivores, pathogens, and mutualists, expanding across trophic levels [8,9].

Plants must endure concurrent climatic and biological stressors that can impact different plant tissues, such as leaves versus roots. Several studies have documented that when aboveground herbivores attack plants, they induce the production of specific phytochemical defenses, such as jasmonic and salicylic acid [10,11,12]. Concurrently, anthropogenic stressors (e.g., sea-level rise and increasing salinity) may influence a completely different tissue type, such as the belowground root system. For example, flooding submerges plant roots and directly limits the diffusion of oxygen to root tissue, disrupting plant physiological and biochemical processes [13,14]. Plants that commonly experience flooding, such as wetland plants, have adapted by allocating resources towards shoot elongation and investing in new leaves to increase oxygen availability to the plant [15,16] or developing aerynchomous tissue that creates air channels in the leaves, stems, and roots of wetland plants, such as *Phragmites* and *Typha* species [17]. However, allocation of resources to growth may come at the cost of defensive traits against herbivory, a tenet of the plant stress hypothesis [18,19]. One expectation is that flooding will cause a reduction in plant resistance traits and consequently render the host plant more susceptible to herbivore damage following flooding events [19]. At present, we know relatively little about whether stressors affecting one plant compartment expose vulnerabilities to other tissue compartments or whether stressors cause similar phytochemical responses across compartments [20,21]. Experimental studies are needed that focus on multiple stressors and more than one type of plant tissue compartment [22,23,24]. Despite their importance, root tissue chemistry remains relatively understudied [21,25,26], highlighting the need for studies that incorporate this crucial tissue compartment.

Plants respond to and defend against climatic stressors and biological invasions through changes in their phytochemical metabolome, yet ecologists have historically focused on a few targeted metabolites when assessing plant responses to stressors [27]. Plants produce a large diversity of metabolites but typically only a few are targeted in quantitative analyses [27,28]. For instance, studies have targeted jasmonic acid and methyl jasmonate as a chemical defense response to herbivory [20,29,30,31], while proline and rutin metabolites are targeted as a response to flooding and drought because they are known to alleviate oxidative stress [32,33]. While targeting metabolites for single stressors has been successful, it is not effective if stressors are concurrent and vastly different [21,34]. An alternative, untargeted approach considers all metabolites and screens for the most influential ones using machine learning techniques [35,36]. Screening all possible metabolites exploits the rich diversity of chemical metabolites and increases the likelihood of finding potent metabolites or their precursors that are effective against one or more stressors (i.e., screening hypothesis) [37,38,39]. While each approach has been influential, a single approach precludes a holistic understanding of plant responses to stress. Comparing the targeted and untargeted approaches is needed to determine the most influential metabolites responsible for protecting plants against concurrent environmental and biological stressors.

Common reed, *Phragmites australis,* provides an ideal model for testing simultaneous stressors on leaf and root phytochemistry. *Phragmites australis* a dominant plant species in freshwater marshes and coastal wetlands [40,41]. In North America, an introduced lineage of *Phragmites* has been displacing native wetland plant species and is ranked as one of the most abundant non-native plants in wetland habitats [42,43]. However, in Louisiana, USA, several phylogenetically distinct lineages of *Phragmites*—both native and non-native—exist along coastal wetlands [44,45,46,47] and are important for stabilizing coastal soils due to their deep root and rhizome structure [48,49]. In the fall of 2016, evidence of *Phragmites* dieback was detected among stands located throughout the Mississippi River Delta (MRD), Plaquemines Parish, Louisiana, USA [50]. Environmental factors, such as interactions among flooding, sea-level rise, drought, and high salinities, and a biotic culprit, a non-native scale insect, *Nipponaclerda biwakoensis* (Kuwana) (Hemiptera: Aclerdidae; hereafter, scale insect), have likely contributed to the dieback [51,52]. The scale insect was discovered at the same time as the dieback, with nearly 100% of stems in the MRD infested at outbreaking levels [50,53]. Interestingly, a previous experimental study demonstrated that the three common *P. australis* lineages in the MRD exhibit differences in their susceptibility to flooding and scale infestation, with the non-native *P. australis* lineage being most resistant compared to the other two lineages [51,53]. Phytochemical trait differences between *P. australis* lineages could explain their difference in susceptibility to these simultaneous stressors, as lineages of *P. australis* are known to differ substantially in their phytochemical diversity [36].

Recently, Cronin and colleagues [51] conducted an experiment to assess the impact of flooding and infestation of an exotic scale insect on aboveground productivity (biomass) of three MRD lineages of *P. australis.* Here, we use both leaf and root tissues collected at the termination of this study to investigate the response of the plant’s metabolome to flooding, scales and the combination of these two stressors. *First*, we used an untargeted analysis that was screened against several reference metabolomic databases to identify known metabolites. *Second*, we used Random Forest, a machine learning statistical technique, to identify the most informative subset of metabolites from the entire metabolome (including metabolites unable to be identified) that predicted flooding and scale infestation. *Third*, using the identified metabolites, we investigated their putative functions—specifically, whether these metabolites are linked to flooding or herbivory based on the published literature. We tested four predictions: (1) Leaf and root tissue have independent responses to each stressor [21,34]. (2) Lineages differ chemically [36], and each lineage employs different strategies in how they respond to abiotic and biotic stressors. Specifically, the non-native lineage is expected to have greater chemical diversity than the other lineages [54,55,56]. (3) Scale-infested or flood-stressed plants will induce higher levels of chemical diversity, suggesting a greater chemical arsenal available to defend against a broad range of plant stressors [37,38,39,57]. (4) Specific metabolites, identified from the literature as putatively associated with flooding [32,33] or herbivory [20,30], exhibit higher concentrations in the flooded and scale-infested plants in comparison to unstressed (control) plants. Overall, these results will help us to understand the phytochemical underpinnings of *P. australis* dieback in the face of anthropogenic stressors.

## 2. Results

(1) Does phytochemical diversity differ between leaves and roots of the same plant and among *P. australis* lineages?

Metabolomic diversity, irrespective of lineage, was significantly higher in leaf tissues (mean ± SE = 7.78 ± 0.001) compared to root (7.39 ± 0.005) tissues (Figure S1; *paired t*-test: *t* = 66.41, *df* = 305, *p* < 0.001), a 5% difference. There was no variation in chemical richness among leaf tissue samples or among root tissues samples. However, leaf tissues had 17% higher mean richness than root tissues (N_LEAF_ = 4394 features VS. N_ROOT_ = 3665 features; the absence of variation in richness within a tissue type made it unnecessary to conduct a statistical test; Figure S1). Chemical evenness was an important component of diversity and varied both within and between tissue types.

(2) Does the chemical diversity of the RF top 10 features vary among *P. australis* lineages?

**Figure 1:**
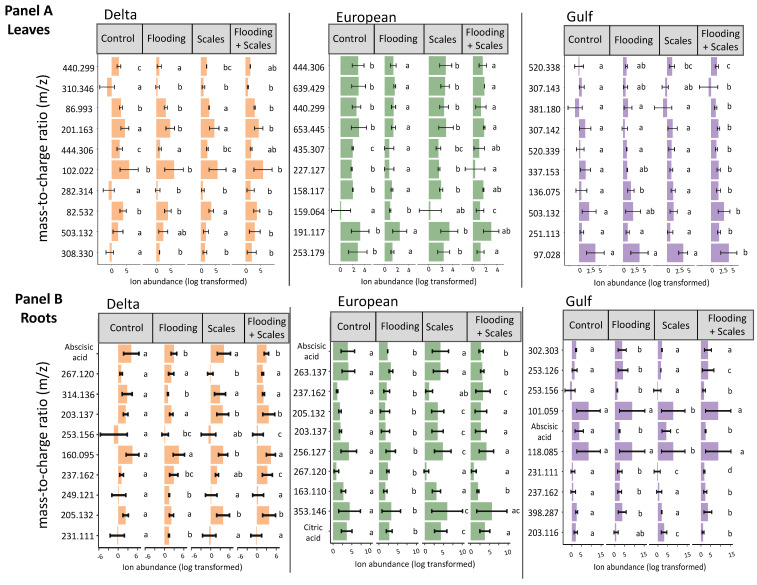
Mean ± SE ion abundance of the top 10 most influential chemical features based on flooding and scale infestation treatments for leaf and root tissues. Random Forest models were run separately for each *P. australis* lineage and tissue type. The treatments consisted of four types: control conditions, flooding, scale infestation, and the combination of flooding and scale infestation. Panels (**A**,**B**) depict the top 10 features for each lineage by leaf and root tissues, respectively. Within lineages, means ± SE are reported and significant differences among treatments are indicated with different letters (*p* ≤ 0.05; based on one-way ANOVA and Tukey pairwise-comparison tests with uncorrected *p* values).

**Figure 2:**
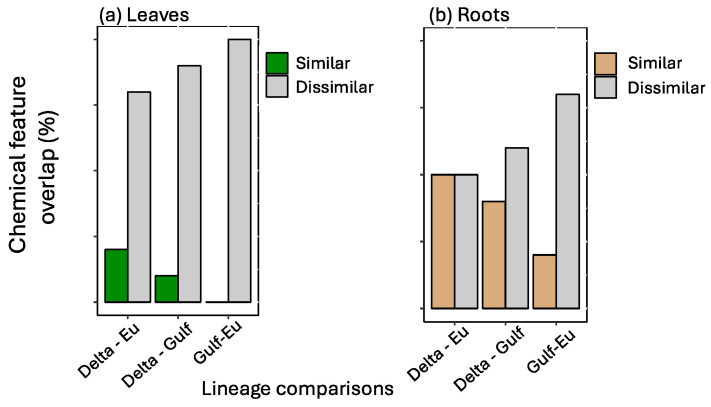
Among pairs of *P. australis* lineages, a comparison of the number of similar and dissimilar top 10 chemical features identified by the Random Forest analysis for (**a**) leaf and (**b**) root tissues. Percent chemical feature overlap is based on the proportion of the top 10 most informative features that are shared between lineages.

The most important features selected by the RF model were unique across tissue type and for each lineage (Figure 1). The features showed variation in average relative abundance across all treatment combinations—flooding, scale infestation, or flooding + scale infestation (Figure 1). Interestingly, for leaf tissues, we found little overlap among lineages in the top 10 chemical features that were most important in distinguishing among stressor treatments (Figure 2). Specifically for leaf tissue, Delta and European lineages shared 20% whereas European and Gulf shared 0% of the same features within the top 10 features identified by RF (Figure 2a). In contrast, there was greater overlap in chemical features among lineages for root tissue, with Delta and European lineages sharing 50% and European and Gulf sharing 20% of the top 10 features identified by RF (Figure 2b). Furthermore, all lineages had the same root feature in common, abscisic acid (Figure 1).

**Figure 3:**
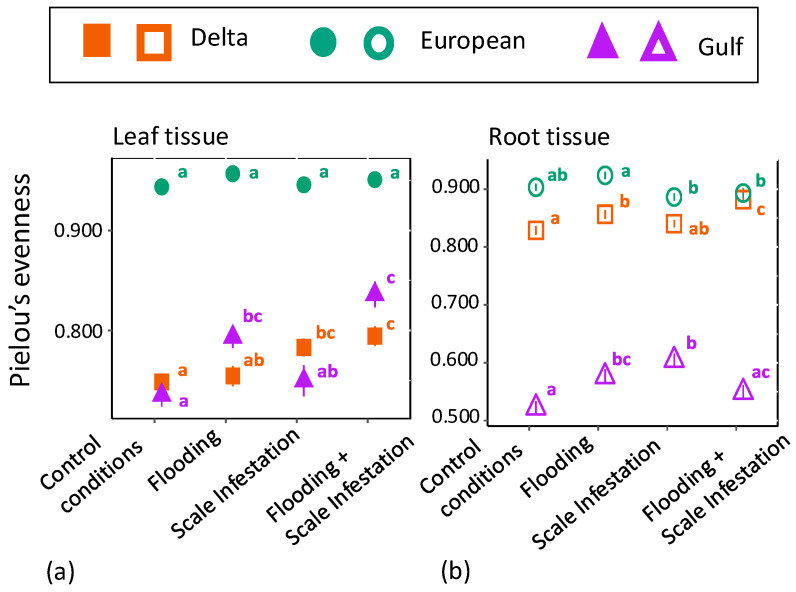
Mean ± SE phytochemical evenness of (**a**) leaf tissue (filled symbols) and (**b**) root tissue (open symbols) for the top 10 most informative features from the entire metabolome as a function of scale infestation and flooding treatment combinations. Pielou’s evenness represents the relative abundance for each metabolite. Significant differences among treatments are indicated with different letters and are only meant for comparison within a lineage (*p* ≤ 0.05; based on one-way ANOVA and Tukey pairwise-comparison tests with uncorrected *p* values).

We also found significant differences in chemical evenness among *P. australis* lineages for the RF top 10 features in both *leaf* (Figure 3a, Table S4; ANOVA likelihood test: *(χ*^2^ = 37.95, *df* = 2, *p* < 0.001) and *root tissue* (Figure 3b, Table S4; ANOVA likelihood test: *(χ*^2^ = 53.81, *df* = 2, *p* = < 0.001) chemistry. For *leaf tissue*, the European lineage had 26% higher chemical evenness compared to Delta and Gulf lineages (Figure 3a, Tukey pairwise-comparison tests with lineage as the only factor; Eu–Delta: estimate = 0.18, *z*-value = 18.32, *p* ≤ 0.001; Eu–Gulf: estimate = −0.17, *z*-value = -17.49, *p* ≤ 0.001), but there was no significant difference in chemical evenness between Delta and Gulf lineages (Figure 3a, Tukey test; Delta–Gulf: estimate = 0.016, *z*-value = 0.715, *p* = 0.754). For *root tissue*, the European lineage had 77% and 11% higher chemical evenness of the 10 most informative features compared to the Gulf and Delta lineages, respectively (Figure 3b, Tukey test; Eu–Gulf: estimate = 0.776, *z*-value = 0.260, *p* < 0.001; Eu–Delta: estimate = 0.111, *z*-value = 6.625, *p* < 0.001).

(3) How does the chemical evenness of the RF top 10 features vary among flooding and scale infestation treatments?

For *leaf tissue* in both Delta and Gulf lineages, plants grown under conditions of scale infestation and flooding had 14% and 7% higher levels of phytochemical evenness compared to plants established in low water depth and low scale densities, respectively (Figure 3a, Table S4; ANOVA likelihood test: *Delta* = *χ*^2^ = 18.64, *df* = 3, *p* < 0.001; *Gulf* = *χ*^2^ = 33.47, *df* = 3, *p* < 0.001). Within the European lineage, the phytochemical evenness of plants was similarly high across all flooding and scale infestation treatments (Figure 3a, Table S4; *χ*^2^ = 5.09, *df* = 3, *p* = 0.17).

For *root tissue,* the Delta lineage grown under scale infestation had 25% lower levels of phytochemical evenness compared to Delta plants grown under control scale densities (Figure 3b, Table S4; ANOVA likelihood test: *χ*^2^ = 30.94, *df* = 3, *p* < 0.001). Within the European lineage, roots grown under flooding conditions had 7% higher levels of phytochemical diversity than those grown under scale infestation (Figure 3b, Table S4; *χ*^2^ = 17.91, *df* = 3, *p* < 0.001). Within the Gulf lineage, roots grown under conditions of a single stressor, either scale infestation or flooding, had 6% and 8% higher levels of phytochemical diversity than plants experiencing both scale infestation and flooding simultaneously (Figure 3b, Table S4; *χ*^2^ = 24.73, *df* = 3, *p* < 0.001).

(4) Does an untargeted or targeted chemical approach provide more insight into plant responses to abiotic and biotic stressors?

From our metabolomic analysis, 238 metabolites were identified (Table S3). Two of those metabolites, abscisic acid and citric acid (found in root tissue only), but none of the seven targeted metabolites that were linked to flooding or herbivory stress in the published literature, appeared in the RF top 10 features (Table 1, Figure 1). Below, we focus on the two metabolites able to be identified because most features selected by the RF model were unable to be identified (Figure 1).

**Figure 4:**
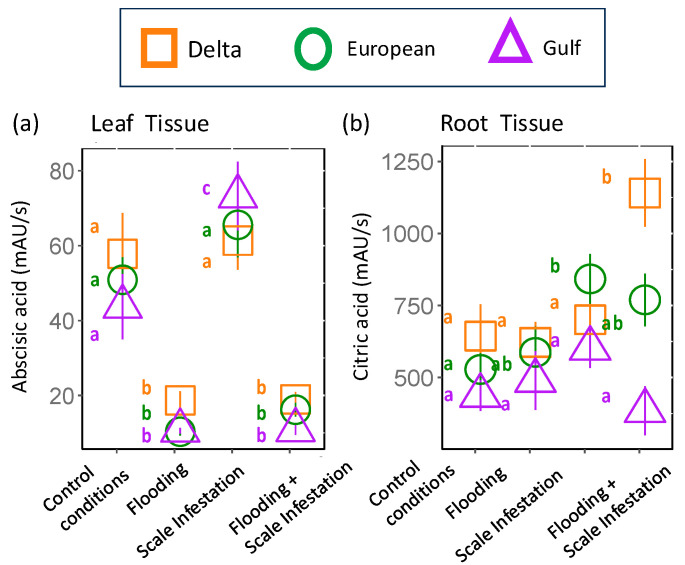
Responses of (**a**) abscisic acid and (**b**) citric acid to stress treatments. These two metabolites were among the top 10 most informative features extracted from the Random Forest model in root tissue only. Each shape represents a distinct lineage (see legend). Within lineages, means ± SE are reported and significant differences among treatments are indicated with different letters (*p* ≤ 0.05; based on one-way ANOVA and Tukey pairwise-comparison tests with uncorrected *p* values).

Identified Metabolites selected by Random Forest: **Abscisic acid** was identified as the most influential root metabolite for Delta and European lineages and one of the top five metabolites for the Gulf lineage (Figure 1). Abscisic acid levels in the roots did not differ among lineages (Figure 4a, Table S4; ANOVA likelihood test: *χ*^2^ = 0.075, *df* = 2, *p* = 0.96). However, all lineages had three-times-higher levels of abscisic acid for plants established in the control and scale infestation treatment combinations compared to plants established in treatments that included flooding (Figure 4a, Table S4; *χ*^2^ = 141.72, *df* = 4, *p* = < 0.001). **Citric acid** in the roots was most strongly affected by flooding and scale infestation (Figure 4b). Delta roots established in flooding + scale infestation treatment had the highest levels of citric acid compared to the other two lineages and had 50% higher levels compared to plants in the control (Figure 4b, Table S4; *χ*^2^ = 16.18, *df* = 3, *p* = 0.001). European roots had 42% higher levels of citric acid for scale-infested plants compared to the control (Figure 4b, Table S4; *χ*^2^ = 16.18, *df* = 3, *p* = 0.001).

**Figure 5:**
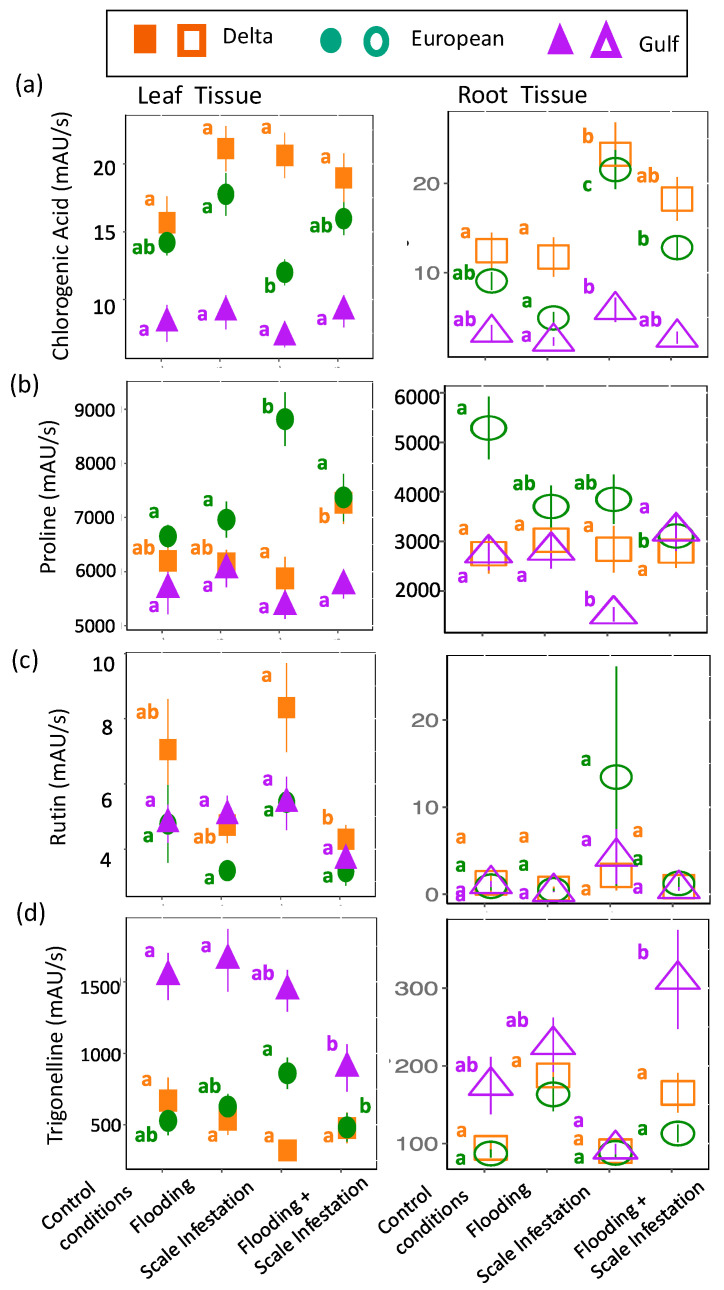
Responses of (**a**) chlorogenic acid, (**b**) proline, (**c**) rutin, and (**d**) trigonelline to flooding stress. Left panel is for leaf tissues (solid symbols) and right panel is for root tissues (open symbols). The relative abundance of each metabolite is in units of mAU/s. Within lineages, means ± SE are reported and significant differences among treatments are indicated with different letters (*p* ≤ 0.05; based on one-way ANOVA and Tukey pairwise-comparison tests with uncorrected *p* values).

*Identified Metabolites with links to flooding and herbivore stressors*: Overall, the seven metabolites that were identified from our untargeted analysis and have known functional links to flooding or herbivory did not respond to our treatments as predicted (see Table 1). Instead, the response of these metabolites to stressors depended on lineage and was complicated by the presence of the other non-targeted stressor. For *flooding*, **chlorogenic acid, proline, rutin, and trigonelline** were predicted to have higher concentrations during instances of flooding stress (Table 1). For leaves, we detected only one treatment effect on **chlorogenic acid** concentration, but it was in the predicted direction. *European leaves* had 30% higher levels of chlorogenic acid for flooded plants compared to scale-infested plants (Figure 5a). Unexpectedly, for roots, an increase in chlorogenic acid was associated with the scale-infested treatment. *Delta and European roots* had 83% and 75% higher levels of chlorogenic acid, respectively, for plants established in scale infestation treatments compared to the control (Figure 5a, Table S5; ANOVA likelihood test: *χ*^2^ = 61.18, *df* = 3, *p* < 0.001). For **proline**, only *European plants* were affected by the stress treatments: *leaves* had a 23% higher concentration for plants infested with scales compared to plants in the control (Figure 5b, Table S5; *χ*^2^ = 9.78, *df* = 2, *p* = 0.008) and *roots* had a 46% higher concentration for plants in control treatments compared to plants experiencing all other combinations of flooding and scale infestation (Figure 5b, Table S5; *χ*^2^ = 8.16, *df* = 2, *p* = 0.017). For **rutin**, only *Delta leaves* responded to our stress treatments. *Delta leaves* had 95% higher rutin levels for plants experiencing scale infestation compared to plants established in the flooding + scale infestation treatment (Figure 5c, Table S5; *χ*^2^ = 18.46, *df* = 3, *p* < 0.001). Finally, for **trigonelline**, *European roots* showed the predicted response with trigonelline levels that were 94% higher for plants established in flooding conditions compared to the control (Figure 5d, Table S5; *χ*^2^ = 30.22, *df* = 3, *p* < 0.001). Interestingly, Gulf tissues exhibited a strong response to the combined effects of flooding and scale insects. *Gulf leaves* had 72% lower trigonelline levels for plants established in the flooding + scale infestation treatment compared to those in the control and flooding treatments (Figure 5d, Table S5; *χ*^2^ = 10.10, *df* = 3, *p* = 0.018), whereas *Gulf roots* had trigonelline levels that were three times higher for plants established in the flooding + scale infestation treatment compared to plants established in the scale infestation treatment only (Figure 5d, Table S5; *χ*^2^ = 30.22, *df* = 3, *p* < 0.001).

**Figure 6:**
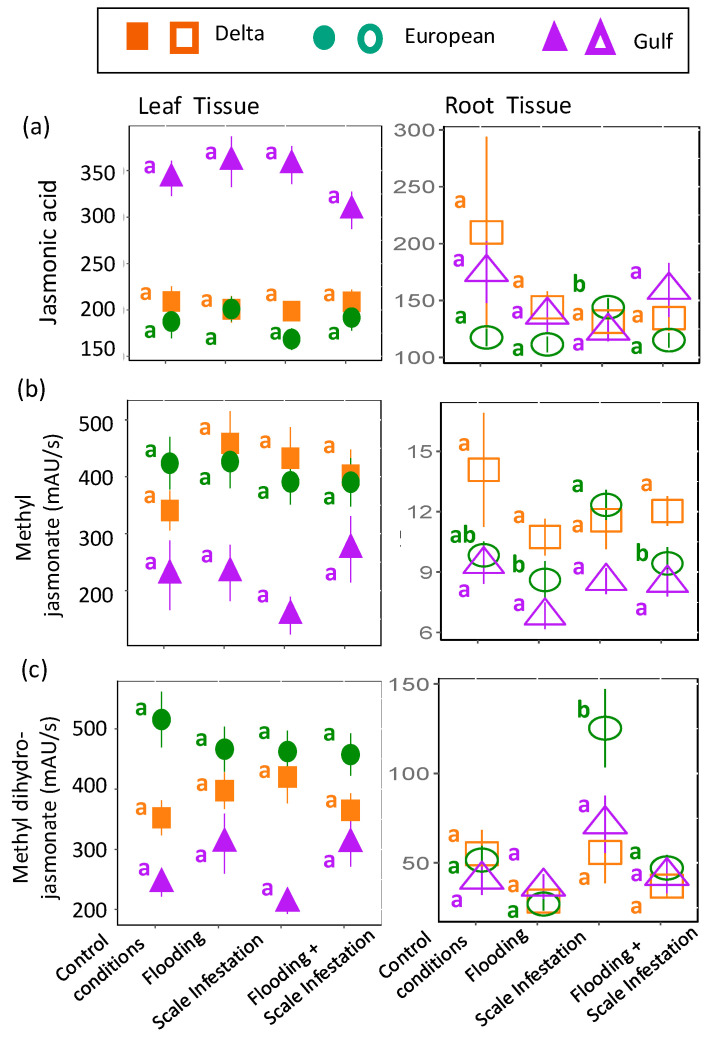
Responses of (**a**) jasmonic acid, (**b**) methyl jasmonate and (**c**) methyl dihydrojasmonate to herbivore stress. Left panel is for leaf tissues (solid symbols) and right panel is for root tissues (open symbols). The relative abundance of each metabolite is in units of mAU/s. Within lineages, significant differences among treatments are indicated with different letters (*p* ≤ 0.05; based on one-way ANOVA and Tukey pairwise-comparison tests with uncorrected *p* values).

The chemical metabolites, **jasmonic acid**, **methyl jasmonate**, and **methyl dihydrojasmonate**, were predicted to increase in plant tissues for treatments involving the addition of scale insects. As before, the response of these compounds to herbivory stress was inconsistent and lineage-dependent. For **jasmonic acid**, there were no treatment effects on leaf jasmonic acid levels, irrespective of lineage (Figure 6a, Table S6; ANOVA likelihood test: *χ*^2^ = 1.92, *df* = 3, *p* = 0.59). In contrast, for *European roots* only, jasmonic acid levels increased by 16% during scale infestation (Figure 6a; Table S6; *χ*^2^ = 14.98, *df* = 3, *p* = 0.02). For **methyl jasmonate**, as predicted, *European roots* induced 44% higher levels of methyl jasmonate for scale-infested plants compared to plants in all flooding treatments (Figure 6b, Table S6; *χ*^2^ = 9.05, *df* = 3, *p* = 0.029). However, across lineages, methyl jasmonate in the leaves was unaffected by treatment (Figure 6b, Table S6; *χ*^2^ = 1.72, *df* = 3, *p* = 0.63). Finally, in support of our prediction, *European roots* induced three times higher levels of **methyl dihydrojasmonate** for plants in the presence of high scale densities compared to all other treatment combinations (Figure 6c, Table S6; *χ*^2^ = 38.88, *df* = 3, *p* < 0.001). There were no treatment effects on leaf methyl dihydrojasmonate (Figure 6c, Table S6; *χ*^2^ = 3.54, *df* = 3, *p* = 0.32).

## 3. Methods

### 3.1. Study System

*Phragmites australis* is a large perennial grass (3–5 m tall) that grows clonally and forms thick monospecific stands, making it one of the most abundant plants to inhabit freshwater and brackish wetlands in North America [58]. In Louisiana, there are three dominant lineages of *P. australis*. One lineage originated from Europe (haplotype M), appeared in North America at least 150 years ago, and has since spread throughout much of the continent (hereafter referred to as the European lineage) [45]. It is considered non-native and a major threat to wetland ecosystems in North America and has proven to be extremely difficult and expensive to manage [59]. However, the European lineage has been noted to provide some beneficial ecosystem services for the Mississippi River Delta (MRD) in mitigating soil erosion [49,50,60]. Another MRD lineage, Delta (haplotype M1), originated from the Mediterranean region of Europe and North Africa and is genetically closely related to the European lineage [46,47]. Delta is reportedly found only in Louisiana [46,47,50]. We classify it as non-native but naturalized. Finally, the Gulf lineage likely expanded its range from Central America and has become established in all southern-most states from Florida to California [45,47,61]. For the purposes of this paper, we refer to Eu as non-native, Delta as naturalized and Gulf as a native lineage.

The MRD is the junction of the Mississippi River with the Gulf of Mexico, making it the seventh largest river delta on Earth [47,62]. Delta is the most common *P. australis* lineage in the MRD, accounting for >90% of the species’ coverage in this area [46,50,53]. The European lineage occurs in the same habitats as the Delta, but stands are typically smaller (<1 ha) and widely dispersed throughout the MRD [46,47,50]. Gulf is also present but typically at higher elevation and in relatively drier habitats. It has low salt tolerance and is rarely found in brackish or saline marshes [63]. 

The MRD has been experiencing a combination of coastal erosion, subsidence, hurricanes, tidal erosion, sea level rise, and human activities. Nineteen-hundred square miles of land loss has occurred since the 1930s, with estimates of another 4120 square miles of land loss over the next 50 years [52]. A new threat to the MRD is the large-scale dieback of *P. australis* [50]. The scale insect that has been implicated as one of the causal agents of *P. australis* dieback in the MRD [50,51,53] is a sap-sucking specialist of *P. australis* and native to Japan, China, and Korea [64,65]. Average mid-summer abundances are 150 scales per meter of stem on the Delta lineage [53,66]. In common-garden studies, even low densities of scales (~20 per m of stem) had a significant negative impact on biomass production in *P. australis* [51,53]. Moreover, in the MRD, scale density is negatively correlated with the normalized difference vegetation index (NDVI), a measure of standing plant biomass [67].

Flooding and sea level rise appears to be another contributor to *P. australis* dieback. Specifically, the marshes in the MRD have experienced an increase in percent time flooded, from 43% in 2007 to 75% in 2022, with monthly maximum flood depth increasing by 1.2 cm yr^−1^ since 2007 [52]. Based on permanent monitoring stations in the MRD (https://www.lacoast.gov/crms, accessed on March 01 2024), Elsey-Quirk and colleagues [52] concluded that the chronic increase in inundation has caused a long-term decline in *P. australis* cover and a reduced capacity for the species to recover from other acute stressors, including salt intrusion and the scale insect.

### 3.2. Experimental Design

A common garden experiment, in which we manipulated *P. australis* lineage, scale presence/absence and inundation level, took place in 2019 over the course of one growing season. The effects of these stressors on plant aboveground biomass, an indicator of plant performance, were reported by Cronin et al. [51], so only a brief description is provided here.

Source populations of *P. australis* have been maintained at Louisiana State University since 2010. By standardizing growing conditions for all source populations, maternal effects that might influence plant growth and chemistry were minimized. In April of 2019, individual rhizomes from three source populations were selected from each of the three lineages (Delta, Gulf and European) and were planted in 16.2 cm diameter pots (18.5 cm tall) filled with peat-based garden soil (see Table S1 for chemical composition of soil). The pots were placed in 1.2 m diameter plastic pools filled with water to a depth of ≈15 cm. Following one week of establishment, each pot was supplemented with 28 g of Osmocote^®^ (9-month, slow-release 15-9-12 NPK). To each pool, we also added a 36 mL solution comprising 45 g of Miracle Gro (24-8-16 NPK, The Scotts Miracle-Gro Company^®^, Marysville, OH, USA), 132 mL of Liquinox^®^ (iron and zinc supplement; Liquinox Co., Orange, CA, USA) and 11.3 L of water.

Gulf and European populations were sourced from Louisiana and other states within their range whereas Delta source populations were necessarily all derived from Louisiana. After five weeks, potted plants were transferred to twenty-four 568 L cattle tanks, with three replicate pots for each source population × lineage combination (27 total pots per tank).

Tanks were randomly assigned to water-depth (low, high) and scale-insect treatments (low and high scale densities). Tanks in the low-water treatment were set at 10 cm water level and those in the high-water treatment were set at 40 cm. The scales were added 7 weeks later when stems were large enough to support scale insects. For plants assigned scale additions, cut *P. australis* stems that were infested with gravid adult female scales were added to the tanks. Hatchling crawlers (first instars) dispersed from the cut stems to the potted experimental plants. It was impossible to prevent scales from infesting the control plants and so these plants were categorized as low-density scale treatment. By the end of the study, October 2019, scale insects in the high-scale-density treatment averaged 20.7 per m of stem, more than five times the density in the low-scale-density treatment [51,68,69]. At the termination of the experiment, we clipped all aboveground stems and leaves, rinsed belowground roots, and obtained separate dry biomass weight for above- versus belowground tissue.

Concurrent with the harvesting of plant biomass, we collected tissues for chemical analysis. Within each tank, we randomly selected one pot with live plant material from each of the nine source populations. We clipped the three uppermost unfurled leaves from as many stems per pot as needed to obtain approximately 100 g wet weight of leaf material. Leaves were wrapped in aluminum foil, dropped into a dewar of liquid nitrogen and flash frozen. At the same time, and from the same pots, approximately 100 g of rhizomes/roots was clipped, washed with tap water to remove soil, wrapped in aluminum foil and flash frozen. Once frozen, both leaves and rhizome/roots were stored in an ice chest with dry ice and later stored in a −80 °C freezer in the laboratory.

### 3.3. Chemical Analysis of Plant Metabolites

*Untargeted approach*: We conducted untargeted chemical analysis on leaf and root samples from our experimental plants (see Table S2 for number of replicates per treatment combination). All leaf and root chemical analyses were performed by Creative Proteomics, NY, USA. In total, 50 mg of ground material was extracted with 800 μL of 80% high-performance liquid chromatography (HPLC)-grade methanol for each leaf and root sample (full extraction protocol can be found in the supplement). Then, 200 μL of the crude extract was dissolved in HPLC-grade methanol with an internal standard of 5 μL of DL-o-Chlorophenylalanine (140 μg/mL). Samples were analyzed by Ultra-High-Performance Liquid Chromatography (UHPLC) using an UltiMate 3000 HPLC combined with a Q Exactive Mass Spectrometer (Thermo Fisher Scientific) and screened with ESI-MS using an ACQUITY UPLC HSS T3 column (100 × 2.1 mm, 1.8 μm) maintained at a temperature of 40 °C. The solvent system employed was HPLC-grade acetonitrile and HPLC-grade water with 0.05% formic acid. The 5 μL injection was eluted at a constant flow of 0.3 mL min^−1^ with a gradient of acetonitrile and water as follows: 0–1.0 min, 95% formic acid/water; 1.0–12.0 min, 95–5% formic acid/water; 12.0–13.5 min, 5% formic acid/water; 13.5–13.6 min, 5–95% formic acid/water; 13.6–16.0 min, 95% formic acid/water. The raw data were corrected for variation associated with instrumentation and extraction errors by calculating the coefficient of variation (CV) using quality control samples. Peaks with CV values greater than 30% represented unreliable measurements and were removed, as described in [70].

*Targeted Approach*: The raw feature data were acquired and aligned using the Compound Discover software (3.0, Thermo Scientific, Waltham, WA, USA) based on the *m*/*z* value and the retention time of the ion signals. Some of the features were identified to specific metabolites (Table S3) using Compound Discover software that uses multiple databases, including mzCloud™, Chemspider™, KEGG, BioCyc, and the Human Metabolome Database (5.0, Wishart Node TMIC, Edmonton, AB, Canada). We were able to identify 268 metabolites out of 4394 features from leaf tissue (6%) and 194 metabolites out of 3665 features from root tissue (5%). We acknowledge that a plant-centric database may have yielded a higher percentage of identified metabolites. However, even when a plant-centric spectral library like Global Natural Product Social Molecular Networking was used with *Phragmites*, only 1.5% of metabolites were identified from untargeted data [36], but also see [71].

### 3.4. Statistical Analyses

Our untargeted metabolomic approach focused on the fragmentation pattern of metabolites—hereafter referred to as features [35,39]. The total numbers of features from the raw data were 4394 from leaf tissue and 3665 from root tissue. Peak areas for each feature in the raw metabolomic dataset were used to compute separate Shannon’s chemical diversities and Pielou’s evenness for leaf and root tissues from each experimental plant [36]. Analyses were performed using the *vegan* package in R version 4.3.1 [72] and using established methods [39,57]. We began by examining the statistical difference in diversity between the entire metabolomic leaf and root chemistries, using a paired *t*-test [72] to account for the likely non-independent chemistries between these tissue compartments within the same plant. Because chemical diversity was significantly different between leaves and roots (see Section 3), all subsequent analyses were conducted on leaf and root tissue separately to gain a comprehensive understanding of the chemical changes occurring within those tissue types.

Next, we used a machine learning approach, Random Forest (RF), to select the top 10 chemical features that are the best predictors of flooding and scale infestation following established methods [36]. Random Forest builds decision trees using randomly chosen variables that best classify observations according to groups and is a method with no prior assumptions about the data [73,74]. We used a total of six separate RF models on the leaf and root data from each of the three lineages—Delta, European, and Gulf—using the *RandomForest* package [75]. Our explanatory variables included all possible stressor treatment combinations classified as follows: (1) control = low scale densities and low water depth; (2) flooding treatment = high water depth and low scale densities; (3) scale infestation = low water depth and high scale densities; (4) flooding + scale infestation treatment = high water depth and high scale densities. We note that RF only allows for one treatment with multiple levels, so it was not possible to construct a model that had the independent and interactive effects of flooding and scale density. In the RF models, our dependent variables were the chemical features. To validate the performance of the model (likelihood of correctly predicting stressor treatment), the data were split into a training set—75% of samples (105 observations)—and test set: the remaining 25% of the samples (35 observations) [76]. The training dataset was used to inform the final model of the number of variables in each tree (mtry = 30) and the number of trees generated (ntree = 5000); the replacement of variables was set to “FALSE”, and we used a stratified sampling scheme to account for an unbalanced number of samples per lineage. For the final model, we used the full dataset and obtained the most important features based on their mean decrease in the Gini coefficient, an index that reflects how much each variable contributes to the homogeneity of decision tree nodes and used to rank the variables [76]. The higher the values of mean decrease in Gini, the more important that feature is in the model used to distinguish stress treatments [73,76]. To estimate model variation, we constructed 500 RF models and calculated the mean and standard deviation. Furthermore, we performed a five-fold cross-validation using the rfcv function in the *RandomForest* package. This is a technique for judging predictive models by evaluating the training model five times using different subsets of the data each time [76]. Ultimately, the RF model ranked the features according to their contribution to the nodes of 5000 decision trees, resulting in the 100 most influential features. We calculated the greatest difference in mean decrease in Gini using the top 100, 50, and 10 features from the RF to assess the fewest metabolites that were able to discriminate amongst the flooding and scale infestation treatments. We found that the top 10 most influential features had the greatest difference in mean decrease in Gini based on our treatment combinations; refer to methods in [36]. Hereafter, we focused on the top 10 features. Using the top 10 features, we calculated chemical evenness using Pielou’s evenness index utilizing the *vegan* package [77].

One of our goals was to determine how effective an untargeted approach is compared to a targeted approach with the aim of adequately assessing the plant’s metabolomic response to simultaneous stressors. The method we used to select our targeted metabolites for flooding and scale infestation is as follows. We searched Web of Science using the key words [metabolite name × flooding or herbivory] using each of the 268 identified metabolites. Next, the metabolites that were linked to flooding or herbivory based on the Web of Science search were kept if they were found in both leaf and root tissue. This search resulted in 7 metabolites that met our criteria.

For flooding stress, **chlorogenic acid, proline, rutin, and trigonelline** were selected (Table 1) [32,78,79]. For herbivory stress, **jasmonic acid**, **methyl jasmonate**, and **methyl dihydrojasmonate** were selected (Table 1) [20,29,30,80].

**Table 1:** Metabolites identified for use in the targeted chemical analysis that are known from the primary literature to be important measures of flooding stress [32,76,77] and herbivory [20,29,30,78] in plants.

Metabolite Name	Metabolite Class	Selected by	Stressor	Function of Metabolite
Abscisic acid	Sesquiterpene	Random Forest	flooding	Abscisic acid accumulation in leaves and roots regulates stomatal closure and osmolyte synthesis, mediating stress response.
Citric acid	Organic acid	Random Forest	flooding	Citric acid accumulation during flooding supports anaerobic respiration and glycolysis, maintaining energy production and pH homeostasis by buffering the acidic environment in plant tissues.
Chlorogenic acid	Carboxylic acid	The literature	flooding	Chlorogenic acid accumulation mitigates stress by regulating water content and oxidative stress.
Proline	Amino acid	The literature	flooding	Proline mitigates flood damage by scavenging reactive oxygen species, regulating redox balance, reducing oxidative stress, and acting as an osmolyte to maintain cell turgor.
Rutin	Flavonoid	The literature	flooding	Rutin accumulation in leaves, fruits, and seeds mediates oxidative damage during drought
Trigonelline	Alkaloid	The literature	flooding	Trigonelline, involved in cell cycle regulation, nodulation, and methylation, is produced during droughts to enhance water storage.
Jasmonic acid	Monocarboxylic acid	The literature	scale infestation	Jasmonic acid is induced by herbivory. Moreover, accumulation of jasmonic acid minimizes water loss by regulating stomatol opening and closing.
Methyl jasmonate	Jasmonate ester	The literature	scale infestation	Methyl jasmonate is induced by herbivory and is a precursor to produce other secondary defenses (i.e., nicotine, caffeoylputrescine).
Methyl dihydro-jasmonate	Jasmonate ester	The literature	scale infestation	Methyl dihydrojasmonate is induced by herbivory and is a precursor to produce other secondary defenses (i.e., nicotine, caffeoylputrescine).

We used a linear mixed-model framework to assess whether flooding and scale infestation treatments had a significant effect on (1) the overall phytochemical diversity of the raw features, (2) the diversity of the top 10 most informative features and (3) the concentration (based on peak height) of targeted stress-indicator metabolites. All analyses were conducted using the *lme4* package [72,81]. We conducted separate mixed-model analyses for each lineage because lineages are known to have unique chemical profiles [36]. All models included a random effect (1|Population/Tank ID) and the fixed effects of four treatment combinations (1—control, 2—flooding conditions, 3—scale infestation, and 4—combined flooding + scale infestation). We chose to keep scale infestation and flooding as combined treatments to make our results comparable with previous analyses. For each model, we confirmed that model residuals were normally distributed. All models were followed by likelihood ratio tests comparing the null model to the treatment model using the *lme4* package [72,81]. Finally, we conducted Tukey multiple comparisons on each analysis to compare levels among treatment combinations using the *multcomp* package [72,82].

## 4. Discussion

Our comprehensive approach, one that examines untargeted (holistic) and targeted plant chemical responses to multiple stressors in both above- and belowground tissues, provides important insights into how plants may chemically respond to threats associated with human activities. Most notably, we found that the phytochemical response strategies, in terms of the chemical diversity and concentration of individual compounds, to environmental stressors was highly context-specific. Chemical diversity (more specifically, evenness) varied among lineages, plant tissue type, and with the kind of stressor, in some cases exhibiting additive, synergistic or antagonistic responses when both stressors occurred together. For example, scale insects induced higher levels of methyl jasmonate in the roots of the European lineage, but when scales were combined with flooding, no induction was evident (an antagonistic response). Second, among the three lineages of *P. australis* in the MRD, the non-native European lineage had the highest levels of constitutive chemical evenness in both the leaves and roots, a trait that could explain its release from the pressures from a wide range of natural enemies found in North America [51,53,83]. Third, the Delta lineage, which is both the dominant lineage in the MRD and most susceptible to dieback, appears to be highly sensitive to both stressors. It induced the highest leaf chemical diversity when scales were abundant and the highest root chemical diversity in response to flooding. The duality of these stressors may be too costly in terms of resources for Delta, particularly in dieback stands, resulting in a moribund plant. Lastly, the literature-based metabolites reputed to be important in a plant’s response to flooding and herbivory did not often respond in the predicted direction. Instead, we found responses to be variable and highly lineage-dependent. Our metabolites used as a proxy for flooding had variable responses. However, our metabolites used as a proxy for herbivory responded as predicted, but only for root tissues of the non-native European lineage. Phytochemical responses that are consistently and strongly lineage-specific is a compelling reason to conduct phytochemical studies at the subspecific level [36]. Below, we discuss the variation in phytochemical responses across *P. australis* lineages to simultaneous stressors and then evaluate a new approach that combines targeted and untargeted analyses at the subspecific level.

### 4.1. Phragmites australis Lineages Use Distinct Strategies to Counter Multiple Stressors

The European lineage outperformed the Delta and Gulf lineages in the production of biomass and had two-fold fewer scales in this experimental study [51]. The varied phytochemical responses across lineages suggest that the best defensive strategy may be to maintain constitutively high levels of chemical diversity. Across lineage comparisons of phytochemical diversity, we found that non-native European plants utilized a constitutive defense strategy by maintaining high levels of chemical diversity. In contrast, the Delta and Gulf plants induced higher levels of chemical diversity depending on the stressors. Induced plant defense is an effective strategy, but if stressors are becoming more intense and frequent, as predicted with global climate change, then a more efficacious strategy would likely be to maintain high levels of constitutive defenses at all times [84,85].

Our results suggest that the phytochemical response to simultaneous stressors is layered, with the first layer being chemical diversity and the second being constitutive versus induced responses. The strategy of maintaining high levels of chemical diversity suggests a better-defended plant, as there is a higher likelihood of having potent compounds to defend against a range of threats (e.g., herbivory, pathogens, drought, and nutrient stress) [39,57,86]. Interestingly, we found that chemical evenness, rather than richness, was driving diversity. This aligns with findings from Salgado and colleagues [36], which included more lineages of *P. australis* and examined plants from both North America and Europe. Both studies suggest that metabolomic pathways are conserved across *P. australis* lineages and that variation in chemical evenness is likely related to environmental adaptation. The other defensive strategy is based on the timing of the plant’s response to stressors, deploying either constitutive (always ready and primed) or induced (activated when the stressor occurs) defenses [87,88]. There are resource and energy trade-offs between constitutive and induced defenses [89]. If the stress is constant, a constitutive defense may be more energy-efficient, while induced defenses might be more pragmatic in cases of occasional stress. For example, European plants generally responded with high constituent levels of chemical diversity, but during scale infestation, plants induced specific metabolites (at least for methyl jasmonate). Our results suggest that constituent and induced defenses work in tandem to provide a constant layer of protection. Future studies should explore whether adaptation to different environmental and biotic pressures over time has conserved constitutive or induced defense strategies among lineages and measure the trade-offs associated.

In our research, we found that stress-induced responses by lineages were context-dependent, indicating that environmental selection and plasticity are pivotal in shaping phytochemical differences among lineages. Delta, European, and Gulf lineages have each adapted to unique environments, influencing their phytochemical responses to abiotic and biotic stressors. For instance, Gulf plants, adapted to higher elevations and drier habitats [63], exhibited high levels of chemical evenness in both leaf and root tissues when subjected to flooding and flooding + scale treatments. This suggests that inducing phytochemicals in response to stress is particularly effective for Gulf plants adapted to occasional flooding environments. In contrast, European and Delta plants, which are found in lower elevations with frequent flooding [47], had higher constitutive levels of chemical evenness in their root tissues compared to Gulf plants. Overall, the phytochemical response of *P. australis* lineages to flooding and scale infestation appears to be linked to their environmental background.

### 4.2. The Costs of Dealing with Multiple Stressors

The metabolomic response of plants to an individual stressor has been extensively studied, but there is relatively little research on the effects of simultaneous stressors across different plant species [90] and metabolomic responses of those species [54]. The few studies that do exist reveal that plants subjected to multiple stressors, such as flooding, herbivory, salinity, and pathogens, experience complex interactions that significantly affect their physiology, growth, and survival [91,92]. One significant challenge is the complex and unpredictable nature of hormonal crosstalk within plants. Plants have evolved intricate signaling networks where various hormones, such as abscisic acid (ABA), jasmonic acid (JA), and salicylic acid (SA), interact to mediate responses to different stressors [93]. These interactions can lead to either synergistic or antagonistic effects, which significantly influence how a plant responds to multiple stresses [93]. We found positive synergistic effects between the flooding and scale infestation stressors, which caused chemical diversity to increase by 12% and 15% in the leaves of Delta and Gulf lineages, respectively, compared to a single stress. Even a small to modest 12–15% change in phytochemical diversity has been shown to be consequential in direct [39,57] and indirect [94,95] plant–insect interactions. Additionally, we observed antagonistic effects between the combined treatments of flooding and scale infestation, whereby flooding stress mitigated the effects of scale infestation in the roots of the Gulf lineage. This could be due to flooding reducing the severity of herbivory by creating an environment less conducive to herbivore survival.

Another difficulty for plants is managing trade-offs in resource allocation when facing multiple stressors. Plants must divide their limited resources between growth, reproduction, and defense [85,96]. This can lead to trade-offs where increasing resistance to one stressor may decrease the plant’s ability to cope with another. For instance, a plant under both drought and pathogen attack may prioritize maintaining oxidative stress at the expense of pathogen resistance [97]. Additionally, plants may face trade-offs between growth and defense, such as the production of secondary metabolites to deter herbivores, which can reduce allocation of resources to growth [98,99].

### 4.3. Environmental Stressors Differentially Affect Chemical Diversity of Leaf and Root Tissue

As indicated in the previous section, leaves and roots did not often respond in concert to the flooding or herbivory stressor treatments, but rather tissue parts were able to compartmentalize their phytochemical responses. For instance, our RF models selected completely different features for leaf and root tissues in terms of the top 10 most informative features relating to flooding and scale infestation across all three lineages. Moreover, chemical evenness did not respond the same for each stressor between leaf and root tissue. For example, flooding was the only stressor that increased evenness in Gulf leaves, whereas scale infestation or flooding stressors alone increased evenness in the roots of Gulf plants. It is difficult to generalize about differences between leaf and root tissues because the three lineages exhibited different phytochemical responses. This may be due to the type of stressor that the specific lineage interacts with and whether this is a constant stress versus a sporadically occurring one. For instance, the defense strategies in milkweed leaves may be evolving independently from root tissue in response to tissue-specific pest stressors [21]. For example, Monarch caterpillars feed elusively on milkweed leaves that induce glycosylated aspecioside cardenolides, whereas the specialized beetle root pest, *Tetraopes tetrophthalmus*, targets milkweed roots which induce syrioside cardenolides [21]. The published literature supports our finding that above- and belowground plant compartments frequently respond independently to different stressors and is consistent with the idea that stressors targeting distinct plant tissues can shape tissue-specific responses, but other stressors may trigger crosstalk between tissue compartments [21]. Our results are unique in that we found metabolomic-level differences between leaves and roots, suggesting that the evolution of plant defense strategies may depend on the environmental adaptations of each lineage, the tissue-specific stress, and whether that stressor is chronic or intermittent.

### 4.4. An Effective Machine Learning Approach to Select Metabolites from the Metabolome

The functionality of specific metabolites can be masked when combining all metabolites into a single metric of diversity [100]. Basically, there is a risk when using the entire untargeted metabolome that the cumulative effects of all metabolites dilute or obscure specific metabolites whose functions are important for mitigating a particular stressor. Although the literature may suggest a functional role of specific metabolites for a specific stressor, our results revealed that those specific metabolites did not respond as predicted by the literature and, in the presence of another stressor, varied among lineages and between tissue types. For flooding, chlorogenic acid, proline, rutin, and trigonelline showed differing patterns in leaves and roots depending on the combination of stressors. Notably, Gulf tissues responded strongly to combined flooding and scale infestation, with significant variations in trigonelline levels across different tissues and treatments. Furthermore, for herbivory, the responses of jasmonic acid, methyl jasmonate, and methyl dihydrojasmonate were tissue-specific, with only root tissue following predictions. European roots showed an increase in jasmonic acid and methyl jasmonate, while methyl dihydrojasmonate levels tripled in high-scale-density treatments. Leaf responses, however, were unaffected by treatments.

Targeting metabolites connected with a single stressor has been used as a proxy for plant stress responses and resilience. However, phytochemical complexity increases with multiple stressors, making the ‘target’ less reliable as other stressors may alter its behavior or influence the plant’s overall response [101,102,103]. This represents a potentially significant gap when targeting specific metabolites based on single stressors. Phytochemical responses are produced by a combination of additive, synergistic, genetic, and environmental stressors [8,9]. The machine learning technique, Random Forest, is an important advancement in untargeted metabolomic studies because it can be used to winnow the data and identify and rank the most informative metabolites based on multiple stressors, regardless of whether the metabolite has a name or not [9,35]. We used this approach and found that the features selected by Random Forest did not match metabolites that were indicated by the literature to function in tolerating flooding stress or herbivory. We also discovered that <1% out of the 238 metabolites identified from public repositories were selected by the Random Forest model as informative features. This low percentage is typical when matching compounds in public repositories [36,104]. The absence of a match should not automatically lead us to remove those features from the dataset, as doing so could mean discarding valuable information. Finally, another benefit to using a machine learning approach is that the model can be tailored to each lineage or species, circumventing the problem of context dependence for species-specific metabolites.

## 5. Conclusions

Our study provides a sobering glimpse into the complex relationship between plant phytochemistry and stressors associated with human activities. Plant chemical responses vary strongly at the intraspecific (genetic) level and among plant compartments depending on the type of stressor and whether stressors occur alone or in combination with other stressors. Our approach was to focus on the whole metabolome, of which only a small fraction of the compounds (≈5%) have been identified. We advocate the use of a machine learning approach such as Random Forest that provides an unbiased and individual-based way to search through a massive database of untargeted chemical features to determine which specific features were the most informative for a given set of plant stressors. This approach is amenable for identifying how plants chemically respond to simultaneous stressors and provides a holistic view regarding how below- and aboveground stressors influence the chemical response in plants.

## Data Availability

This research has not been published elsewhere. Data are available in Figshare data repository (https://doi.org/10.6084/m9.figshare.27304323 accessed on 10 September 2020).

## Supplementary Materials

The following supporting information can be downloaded at: https://www.mdpi.com/article/10.3390/plants13223133/s1, Table S1: Chemical composition of peat-based garden soil; Table S2: Replication number for each treatment combination; Figure S1: Overall chemical diversity between leaves and roots of Delta, EU, and Gulf lineages; Table S3: List of identified metabolites and associated compound class; Table S4: Summary statistics for metabolites selected by Random Forest; Table S5: Summary statistics for metabolites targeted for flooding; Table S6: Summary statistics for metabolites targeted for herbivory.
