# Supplementary material for: The Effects of Anthropogenic Stressors on Above- and Belowground Phytochemical Diversity of the Wetland Grass, Phragmites australis
Source: Plants (Basel). 2024 Nov 7;13(22):3133. doi: 10.3390/plants13223133 (PMC11597171; doi:10.3390/plants13223133)
Supplement: Supplementary file 1 [file plants-13-03133-s001.zip › plants-3210019-supplementary.pdf]

The effects of anthropogenic stressors on above- and below-ground phytochemical diversity of the wetland grass, *Phragmites australis*

Andrea Glassmire[1], Ana Salgado[1], Rodrigo Diaz[2], Joseph Johnston[1], Joshua S. Snook[2], Laura A. Meyerson, and James T. Cronin[1]

SUPPLEMENTARY

Table of Contents

Table S1. Chemical composition of peat-based garden soil

Table S2. Replication number for each treatment combination

Fig S1. Overall chemical diversity between leaves and roots of Delta, EU, and Gulf lineages

Table S3. List of identified metabolites and associated compound class

Table S4. Summary statistics for metabolites selected by Random Forest

Table S5. Summary statistics for metabolites targeted for flooding

Table S6. Summary statistics for metabolites targeted for herbivory

Table S1. Chemical composition of the peat-based garden soil. Average values based on three samples of soil.

pH	% Organic Matter	Carbon %	Nitrogen %
6.58	6.77	8.48	0.20

Phosphorus (ppm)	Potassium (ppm)	Calcium (ppm)	Magnesium (ppm)
41.48	255.93	3014.84	277.23

Sulfur (ppm)	Zinc (ppm)	Iron (ppm)	Copper (ppm)
6.99	9.38	128.52	4.28

Sodium (ppm)	Aluminum (ppm)	Manganese (ppm)
15.39	0.89	58.47

Table S2. The replication number for each treatment combination based on lineage. We conducted untargeted chemical analysis on leaf and root samples from three lineages of *P. australis* grown under flooding and scale infestation.

Lineage	Tissue Type	Flooding	Scales	Total Reps
Delta	Leaves	low	scales	32
Delta	Leaves	low	no scales	30
Delta	Leaves	high	scales	30
Delta	Leaves	high	no scales	29
EU	Leaves	low	scales	35
EU	Leaves	low	no scales	34
EU	Leaves	high	scales	36
EU	Leaves	high	no scales	36
Gulf	Leaves	low	scales	30
Gulf	Leaves	low	no scales	31
Gulf	Leaves	high	scales	30
Gulf	Leaves	high	no scales	26
Delta	Roots	low	scales	25
Delta	Roots	low	no scales	20
Delta	Roots	high	scales	26
Delta	Roots	high	no scales	29
EU	Roots	low	scales	32
EU	Roots	low	no scales	29
EU	Roots	high	scales	28
EU	Roots	high	no scales	30
Gulf	Roots	low	scales	27
Gulf	Roots	low	no scales	24
Gulf	Roots	high	scales	24
Gulf	Roots	high	no scales	24

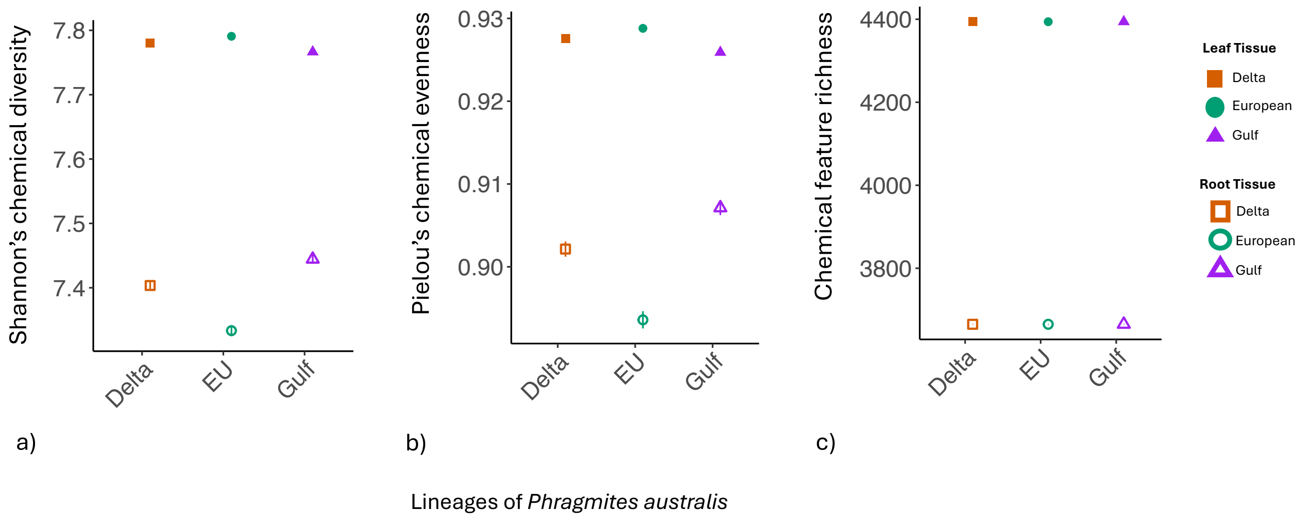

Fig S1. Overall chemical diversity between leaves and roots of the same plant. Chemical diversity was calculated using the entire raw feature data (4,394 features from leaf tissue and 3,665 features from root tissue). (A) Shannon's chemical diversity, (B) chemical evenness, and (C) chemical richness. Solid shapes represent leaf chemistry and outline shapes represent root chemistry. The color of the shape represents lineage (orange = Delta, green = European, purple = Gulf). Symbols with bars represent mean ± SE. In some cases, the SE bars are too small to be evident in these plots.

Table S3. List of identified metabolites and associated compound class across leaf and root tissues. The raw feature data were acquired and aligned using the Compound Discover software based on the m/z value and the retention time of the ion signals. Only a fraction of the features was identified to specific metabolites using Compound Discover software.

Compound name	Compound class
Acetamidobutanoic acid	Amino Acid
Adenine	Purine
Adenosine	Nucleoside
Adrenic acid	Polyunsaturated fatty acid
Allantoic acid	Amino acid
Allantoin	Imidazole
Aminoacrylic acid	Amonocarboxylic acid
Aminocyclopropanecarboxylic acid	Carboxylic acid
Anofinic acid	Organic Acid
Arachidonic acid	Fatty acid
Aspartylphenylalanine	Peptide
Benzoic acid	Carboxylic acid
Chenodeoxycholic acid	Carboxylic acid
Chlorogenic acid	Caffeoylquinic acid
Cinnamic acid	Carboxylic acid
Citric acid	Organic Acid
Citrulline	Amino acid
Coumaperine	Alkaloid
Cytidine	Nucleoside
D Alanyl D alanine	Peptide
D Xylose	Monosaccharide - aldose
Delphinidin-3-6-malonyl glucoside	Flavonoid - anthocyanin
Delphinidin-3-glucoside	Flavonoid - anthocyanin
Deoxymugineic acid	Carboxylic acid
Dethiobiotin	Vitamin Derivative
Diaminopimelic acid	Amino Acid
Dibutyl malate	Carboxylic acid
Diethyl phthalic acid	Phthalic Acid Derivative
Dihydroferulic acid	Phenolic Acid
Dihydroferuperine	Alkaloid
Dihydrolipoamide	Co-enzyme
DL-o-Chlorophenylalanine	Amino Acid
Docosahexaenoic acid	Omega-3 fatty acid
Docosapentaenoic acid	Omega-3 fatty acid
Estrone	Steriod Hormone
Ferulic acid	Hydroxycinnamic acids
Furancarboxaldehyde	Aldehyde
gamma Glutamyltyrosine	Peptide
Glucose	Monosaccharide
Glucose-1-phosphate	Carbohydrate Derivative
Glutamylleucine	Peptide
Glyceraldehyde-3-phosphate	Aldotriose phosphate
Guanidinobutanoic acid	Organic acid

Table S3 *continued…*

Na-p-Hydroxycoumaroyltryptophan	Amino acid
Naphthol	Naphthol
Neopterin	Pteridine Derivative
Nicotinic acid	Carboxylic acid
Oleoylethanolamide	Ethanolamide
Ornithine	Amino acid
Oxo-4-methylthiobutanoic acid	Fatty acid
Oxododecanoic acid	Fatty Acid
Oxoglutaric acid	Gamma-keto acids
p Cresol	Phenol
Phenylacetaldehyde	Aldehyde
Phenylacetic acid	Monocarboxylic acid
Phosphate	Anion, salt
Phosphoglycolic acid	Monoalkyl phosphate
Piperidine	Alkaloid
Porphobilinogen	Pyrrole Derivative
Proline	Amino acid
Propionic acid	Carboxylic acid
Putrescine	Diamine
Pyridoxic acid	Pyridinecarboxylic acid
Pyridoxine	Pyridoxine (vitamin b6)
Pyroglutamic acid	Amino acid
Quercetin	Flavonoid - flavonol
Quercetin-3,6-malonyl glucoside	Flavonoid glycoside
Quercetin-4-glucoside	Flavonoid glycoside
Quercitrin	Flavonoid glycoside
Quinic acid	Organic Acid
Riboflavin	Vitamin B 2
Rosmaricine	Diterpene lactone
Rutin	Disaccharide derivative
Rutin 1	Organic compound
S Cysteinosuccinic acid	Amino acid
Sebacic acid	Dicarboxylic Acid
Sinapic acid	Hydroxycinnamic acids
Spermidine	Polyamine
Stearidonic acid	Fatty Acid
Styrene	Vinylarene
Tetrahydropersin	Fatty alcohol
Thiamine	Vitamin B-1
Threonic acid	Sugar acid
Thymidine	Nucleoside
Thymine	Pyrimidine nucleobase
Thymol	Phenol - monoterpene
Traumatin	Plant Hormone

Table S3 *continued…*

Guanosine	Purine
Histamine	Organic compound
Homo L arginine	Amino acid
Homomethionine	Amino acid
Hydroxy L tryptophan	Amino acid
Hydroxycitrulline	Amino Acid
Hydroxymelatonin	Neurotransmitter Derivative
Hydroxyprolyl Leucine	Amino acid
Indole-3-carboxaldehyde	Indole alkaloid
Indoleacetaldehyde	Indole
Indoleacrylic acid	Carboxylic acid
Isorhamnetin	Flavonoid
Itaconic acid	Carboxylic acid
L Alanine	Amino acid
L Aspartic acid	Amino acid
L Coprine	Alkaloid
L Formylkynurenine	Amino acid
L Glutamic acid	Carboxylic acid
L Histidine	Amino acid
L Histidinol	Amino alcohol
L Methionine	Amino acid
L Methionine sulfoxide	Amino acid
L Phenylalanine	Amino acid
L Serine	Amino acid
L Theanine	Amino acid
L Threonine	Amino acid
Levulinic acid	Carboxylic acid
Linolenic acid	Fatty acid
Lipoamide	Co-enzyme
Mannitol	Sugar alcohol
Melatonin	Neurotransmitter Derivative
Meprobamate	Carbamate
Methoxycinnamic acid	Cinnamic acid
Methoxydimethyltryptamine	Tryptamine Derivative
Methyl-2-oxovaleric acid	Carboxylic acid
Methyladenine	Purine
Methylphenidate	Central Nervous System Stimulant
Methylthioadenosine	Nucleoside
Muramic acid	Amino Sugar
Murrayanol	Alkaloid
Myrianthic acid	Triterpene acid
N Acetylserotonin	Neurotransmitter Derivative
N Undecanoylglycine	Fatty Acid
N1, N10-Dicoumaroylspermidine	Polyamine conjugates
N8 Acetylspermidine	Amino acid

Table S3 *continued…*

Trigonelline	Alkaloid
Tryptophan	Amino Acid
Tryptophol	Indolyl alcohol
Undecylenic acid	Unsaturated fatty acid
Uracil	Pyrimidine nucleobase
Uridine	Nucleoside
Urocanic acid	Carboxylic acid
Xanthine	Purine

Table S4. Summary statistics for metabolites selected by Random Forest. The estimate, standard error, *df*, *T*-value, and *P*-value columns represent the summary stats from the linear mixed model. The final three columns that include χ^2, *df*, and *P*-value represent the statistics from the ANOVA likelihood test that compared the null model to the linear mixed model.

Analysis	response	predictors	Estimate	Std. Error	df	Tvalue	Pvalue	χ2	df	Pvalue
**Question 1: Overall Chemical Diversity between Leaves and Roots**	**Chemcial**	Intercept (Delta lineage)	9.28E-01	1.20E-03	3.28E+01	776.029	<2e-16***			
	**Evenness of**	European lineage	-3.28E-03	1.10E-03	5.98E+00	-2.984	0.0246*			
	**Leaves & Roots**	Gulf lineage	1.39E-03	1.12E-03	6.46E+00	1.234	0.2603			
	**Combined**	Rhizome tissue	-2.70E-02	5.90E-04	6.86E+02	-45.786	<2e-16***			
	All features	Replicates	2.60E-04	5.58E-04	6.89E+02	0.467	0.6408			
	Likelihood ratio test	intercept of null model	9.14E-01	1.88E-03	1.98E+02	485.204	<2e-16***	976.2	3	< 2.2e-16 ***
	**Chemcial**	Intercept (Delta lineage)	9.28E-01	8.29E-04	1.02E+01	1.12E+03	<2e-16 ***			
	**Evenness of**	European lineage	1.11E-03	1.03E-03	6.00E+00	1.09E+00	0.319			
	**Leaves Only**	Gulf lineage	-1.75E-03	1.03E-03	6.14E+00	-1.69E+00	0.141			
	All features	Replicates	1.33E-05	2.58E-04	3.70E+02	5.20E-02	0.959			
	0									
	Likelihood ratio test	intercept of null model	9.28E-01	6.80E-04	1.83E+01	1364.035	<2e-16 ***	7.53	2	0.05
	**Chemcial**	Intercept (Delta lineage)	9.01E-01	2.24E-03	2.55E+01	402.869	< 2e-16 ***			
	**Evenness of**	European lineage	-8.56E-03	2.20E-03	6.10E+00	-3.894	0.00779 **			
	**Roots Only**	Gulf lineage	5.05E-03	2.24E-03	6.53E+00	2.259	0.06116 .			
	All features	Replicates	6.58E-04	1.06E-03	3.09E+02	0.623	0.53399			
	Likelihood ratio test	intercept of null model	9.00E-01	2.67E-03	1.91E+01	337.686	<2e-16 ***	18.21	2	0.00011***
**Question 2: How does chemical diversity vary among lineages?**	**Chemical**	Intercept (Delta lineage)	7.55E-01	1.09E-02	3.16E+01	6.95E+01	< 2e-16 ***			
	**Evenness of**	European lineage	1.78E-01	9.94E-03	5.75E+00	1.79E+01	2.92e-06 ***			
	**Leaves Only**	Gulf lineage	7.12E-03	1.02E-02	6.27E+00	6.99E-01	0.5099			
	Top 10 features	Replicates	1.06E-02	5.32E-03	3.71E+02	2.00E+00	0.0464 *			
	selected by									
	Random Forest									
	Likelihood ratio test	intercept of null model	1.10E-02	5.32E-03	3.69E+02	2.06E+00	0.0402 *	37.95	2	5.73e-09***
	**Chemical**	Intercept (Delta lineage)	8.54E-01	9.54E-03	5.89E+01	8.96E+01	< 2e-16 ***			
	**Evenness of**	European lineage	4.82E-02	7.29E-03	5.62E+00	6.61E+00	0.000755 ***			
	**Roots Only**	Gulf lineage	-2.89E-01	7.57E-03	6.40E+00	-3.82E+01	8.58e-09 ***			
	Top 10 features	Replicates	-6.04E-04	5.27E-03	3.10E+02	-0.115	0.908855			
	selected by									
	Random Forest									
	Likelihood ratio test	intercept of null model	7.75E-01	5.31E-02	8.37E+00	1.46E+01	3.07e-07 ***	53.81	2	2.07e-12***
**Question 3: How does chemical diversity vary among flooding and scale stressors in *leaves*?**	***Delta***	intercept (Low Scales & Low Water)	7.28E-01	1.91E-02	1.46E+01	3.80E+01	4.91e-16 ***			
	**Chemical**	Low Scales & High Water Depth	9.06E-03	1.23E-02	1.14E+02	7.35E-01	0.463571			
	**Evenness of**	High Scales & Low Water Depth	3.49E-02	1.20E-02	1.14E+02	2.91E+00	0.004355 **			
	**Leaves Only**	High Scales & High Water Depth	4.69E-02	1.22E-02	1.14E+02	3.851	0.000194 ***			
	Top 10 features	Replicates	1.38E-02	8.37E-03	1.14E+02	1.648	0.102042			
	selected by									
	Random Forest									
	Likelihood ratio test	intercept of null model	7.52E-01	1.81E-02	1.06E+01	41.615	4.1e-13 ***	18.64	3	0.00032***
	European	intercept (Low Scales & Low Water)	9.44E-01	7.83E-03	1.36E+02	1.21E+02	<2e-16 ***			
	**Chemical**	Low Scales & High Water Depth	1.32E-02	6.45E-03	1.36E+02	2.04E+00	0.0432 *			
	**Evenness of**	High Scales & Low Water Depth	2.15E-03	6.50E-03	1.36E+02	3.30E-01	0.7418			
	**Leaves Only**	High Scales & High Water Depth	7.22E-03	6.44E-03	1.36E+02	1.12	0.2646			
	Top 10 features	Replicates	-4.88E-04	4.21E-03	1.36E+02	-0.116	0.9079			
	selected by									
	Random Forest									
	Likelihood ratio test	intercept of null model	9.50E-01	6.91E-03	1.39E+02	137.464	<2e-16 ***	5.09	3	0.17
	Gulf	intercept (Low Scales & Low Water)	7.11E-01	2.15E-02	1.12E+02	3.31E+01	< 2e-16 ***			
	**Chemical**	Low Scales & High Water Depth	5.72E-02	1.85E-02	1.12E+02	3.09E+00	0.0025 **			
	**Evenness of**	High Scales & Low Water Depth	1.41E-02	1.78E-02	1.12E+02	7.91E-01	0.4305			
	**Leaves Only**	High Scales & High Water Depth	9.87E-02	1.79E-02	1.12E+02	5.527	2.15e-07 ***			

Table S4 continued

	Top 10 features	Replicates	1.66E-02	1.18E-02	1.12E+02	1.407	0.1621			
	selected by									
	Random Forest									
	Likelihood ratio test	intercept of null model	0.74345	0.0217	115	34.267	<2e-16 ***	33.47	3	2.57E-07
**Question 3: How does chemical diversity vary among flooding and scale stressors? in *roots* ?**	***Delta***	intercept (Low Scales & Low Water)	8.36E-01	1.33E-02	3.61E+01	6.28E+01	< 2e-16 ***			
	**Chemical**	Low Scales & High Water Depth	2.54E-02	9.39E-03	9.39E+01	2.71E+00	0.00798 **			
	**Evenness of**	High Scales & Low Water Depth	8.61E-03	9.75E-03	9.46E+01	8.83E-01	0.37967			
	**Roots Only**	High Scales & High Water Depth	5.05E-02	9.61E-03	9.39E+01	5.261	8.99e-07 ***			
	Top 10 features	Replicates	-3.77E-03	6.43E-03	9.32E+01	-0.586	0.55929			
	selected by									
	Random Forest									
	Likelihood ratio test	intercept of null model	0.862378	0.013073	21.499838	65.965	<2e-16 ***	30.94	3	8.74E-07
	European	intercept (Low Scales & Low Water)	9.16E-01	1.26E-02	4.50E+01	7.28E+01	<2e-16 ***			
	**Chemical**	Low Scales & High Water Depth	2.05E-02	9.47E-03	1.12E+02	2.17E+00	0.0322 *			
	**Evenness of**	High Scales & Low Water Depth	-1.79E-02	9.33E-03	1.12E+02	-1.92E+00	0.0571 .			
	**Roots Only**	High Scales & High Water Depth	-1.05E-02	9.62E-03	1.12E+02	-1.087	0.2794			
	Top 10 features	Replicates	-8.20E-03	6.46E-03	1.13E+02	-1.27	0.2068			
	selected by									
	Random Forest									
	Likelihood ratio test	intercept of null model	0.913343	0.01133	51.349128	80.61	<2e-16 ***	17.91	3	4.58E-04
	Gulf	intercept (Low Scales & Low Water)	5.07E-01	2.02E-02	9.40E+01	2.52E+01	< 2e-16 ***			
	**Chemical**	Low Scales & High Water Depth	5.28E-02	1.71E-02	9.40E+01	3.09E+00	0.00267 **			
	**Evenness of**	High Scales & Low Water Depth	8.17E-02	1.66E-02	9.40E+01	4.92E+00	3.7e-06 ***			
	**Roots Only**	High Scales & High Water Depth	2.64E-02	1.71E-02	9.40E+01	1.542	0.12632			
	Top 10 features	Replicates	1.06E-02	1.11E-02	9.40E+01	0.954	0.34263			
	selected by									
	Random Forest									
	Likelihood ratio test	intercept of null model	0.5468	0.01957	97	27.934	<2e-16 ***	24.73	3	1.76E-05
**Question 4: Compare untargeted data to targeted data selected by the litearture**	***Abscisic acid***	intercept (Low Scales & Low Water)	4.91E+01	6.31E+00	2.49E+02	7.78E+00	1.99e-13 ***			
	**Roots Only**	Low Scales & High Water Depth	-3.69E+01	4.98E+00	3.10E+02	-7.42E+00	1.17e-12 ***			
	selected by	High Scales & Low Water Depth	1.65E+01	4.97E+00	3.11E+02	3.32E+00	0.00101 **			
	Random Forest	High Scales & High Water Depth	-3.49E+01	5.05E+00	3.09E+02	-6.9	2.95e-11 ***			
		Replicates	8.26E-01	3.35E+00	3.08E+02	0.246	0.80574			
	Likelihood ratio test	intercept of null model	36.494	2.184	9.493	16.71	2.34e-08 ***	141.72	4	< 2.2e-16 ***
	Citric acid	intercept (Low Scales & Low Water)	4.66E+02	1.04E+02	7.43E+01	4.50E+00	2.46e-05 ***			
	**Roots Only**	Low Scales & High Water Depth	3.19E+01	7.18E+01	3.06E+02	4.44E-01	0.65761			
	selected by	High Scales & Low Water Depth	1.97E+02	7.18E+01	3.07E+02	2.74E+00	0.00646 **			
	Random Forest	High Scales & High Water Depth	2.38E+02	7.28E+01	3.06E+02	3.27	0.00120 **			
		Replicates	4.17E+01	4.83E+01	3.06E+02	0.864	0.38813			
	Likelihood ratio test	intercept of null model	589.81	93.59	53	6.302	5.97e-08 ***	16.18	3	0.0010 **

Table S5. Summary statistics for metabolites targeted for flooding. The estimate, standard error, *df*, *T*-value, and P-value columns represent the summary stats from the linear mixed model. The final three columns that include χ^2, *df*, and *P*-value represent the statistics from the ANOVA likelihood test that compared the null model to the linear mixed model.

response	predictors	Estimate	Std. Error	df	Tvalue	Pvalue	χ2	df	Pvalue
Chlorogenic acid	intercept (Low Scales & Low Water, Delta Lineage)	1.52E+01	2.10E+00	5.12E+01	7.24E+00	2.28e-09 ***			
**Leaves Only**	Low Scales & High Water Depth	5.64E+00	2.02E+00	3.61E+02	2.79E+00	0.00558 **			
selected by	High Scales & Low Water Depth	4.91E+00	1.97E+00	3.60E+02	2.489	0.01325 *			
the literature	High Scales & High Water Depth	3.32E+00	2.00E+00	3.60E+02	1.659	0.09792 .			
	European lineage	-1.6302	2.4004	2.29E+01	-6.79E-01	5.04E-01			
	Gulf lineage	-7.4549	2.4376	24.1967	-3.058	0.00537 **			
	Replicates	3.63E-01	7.48E-01	3.62E+02	4.85E-01	0.628			
	Low Scales & High Water Depth : EU Lineage	-2.05E+00	2.74E+00	3.61E+02	-0.746	0.456			
	High Scales & Low Water Depth : EU Lineage	-7.0281	2.7168	360.6876	-2.587	0.01008 *			
	High Scales & High Water Depth : EU Lineage	-1.5565	2.7286	360.3611	-0.57	0.56875			
	Low Scales & High Water Depth : Gulf Lineage	-4.7749	2.8892	360.7159	-1.653	0.09926 .			
	High Scales & Low Water Depth : Gulf Lineage	-5.9234	2.797	360.3469	-2.118	0.03488 *			
	High Scales & High Water Depth : Gulf Lineage	-2.5199	2.8214	360.2323	-0.893	0.37238			
Likelihood ratio test	intercept of null model	13.8017	2.0451	17.3373	6.749	3.07e-06 ***	39.41	11	4.507e-05 ***
Chlorogenic acid	intercept (Low Scales & Low Water, Delta Lineage)	9.25E+00	2.89E+00	5.54E+01	3.21E+00	0.00223 **			
**Roots Only**	Low Scales & High Water Depth	3.72E-01	2.66E+00	3.02E+02	1.40E-01	0.88867			
selected by	High Scales & Low Water Depth	1.22E+01	2.76E+00	3.04E+02	4.437	1.28e-05 ***			
the literature	High Scales & High Water Depth	6.84E+00	2.72E+00	3.02E+02	2.517	0.01235 *			
	European lineage	-2.5921	3.2611	2.47E+01	-7.95E-01	4.34E-01			
	Gulf lineage	-8.1275	3.3551	27.3659	-2.422	0.02230 *			
	Replicates	1.55E+00	9.84E-01	3.00E+02	1.58E+00	0.11628			
	Low Scales & High Water Depth : EU Lineage	-4.26E+00	3.56E+00	3.01E+02	-1.199	0.23133			
	High Scales & Low Water Depth : EU Lineage	0.3101	3.6115	302.7936	0.086	0.93162			
	High Scales & High Water Depth : EU Lineage	-2.9494	3.6266	300.8194	-0.813	0.41671			
	Low Scales & High Water Depth : Gulf Lineage	-1.5198	3.7324	300.8462	-0.407	0.68415			
	High Scales & Low Water Depth : Gulf Lineage	-9.7798	3.7565	302.5711	-2.603	0.00969 **			
	High Scales & High Water Depth : Gulf Lineage	-7.4692	3.7744	300.7273	-1.979	0.04874 *			
Likelihood ratio test	intercept of null model	8.664	2.606	22.141	3.325	0.00305 **	97.38	11	5.883e-16 ***
Proline	intercept (Low Scales & Low Water, Delta Lineage)	6.15E+03	5.84E+02	3.05E+01	1.05E+01	1.1e-11 ***			
**Leaves Only**	Low Scales & High Water Depth	3.17E+01	5.13E+02	3.61E+02	6.20E-02	0.95076			
selected by	High Scales & Low Water Depth	-3.02E+02	4.99E+02	3.60E+02	-0.605	0.54574			
the literature	High Scales & High Water Depth	1.11E+03	5.07E+02	3.60E+02	2.179	0.02997 *			
	European lineage	464.29	694.92	1.57E+01	6.68E-01	5.14E-01			
	Gulf lineage	-479.53	703.27	16.4	-0.682	0.50485			
	Replicates	-3.63E+00	1.90E+02	3.61E+02	-1.90E-02	0.98475			
	Low Scales & High Water Depth : EU Lineage	3.19E+02	6.96E+02	3.61E+02	0.459	0.64683			
	High Scales & Low Water Depth : EU Lineage	2578.99	688.64	360.54	3.745	0.00021 ***			
	High Scales & High Water Depth : EU Lineage	-334.37	691.57	360.33	-0.484	0.62903			
	Low Scales & High Water Depth : Gulf Lineage	334.63	732.33	360.56	0.457	0.64799			
	High Scales & Low Water Depth : Gulf Lineage	-11.1	708.89	360.33	-0.016	0.98752			
	High Scales & High Water Depth : Gulf Lineage	-1032.46	715.07	360.26	-1.444	0.14965			
Likelihood ratio test	intercept of null model	6465.92	439.02	28.55	14.728	7.18e-15 ***	46.42	11	2.728e-06 ***
Proline	intercept (Low Scales & Low Water, Delta Lineage)	3.29E+03	6.58E+02	5.60E+01	4.99E+00	6.19e-06 ***			
**Roots Only**	Low Scales & High Water Depth	3.11E+02	6.04E+02	3.02E+02	5.16E-01	0.60651			
selected by	High Scales & Low Water Depth	1.99E+02	6.27E+02	3.04E+02	0.318	0.75098			
the literature	High Scales & High Water Depth	6.29E+01	6.17E+02	3.02E+02	0.102	0.91896			
	European lineage	2634.54	745.39	2.51E+01	3.53E+00	0.00161 **			

Table S5 continued

	Gulf lineage	76.01	766.66	27.87	0.099	0.92174			
	Replicates	-3.97E+02	2.24E+02	3.00E+02	-1.78E+00	0.07693 .			
	Low Scales & High Water Depth : EU Lineage	-1.95E+03	8.08E+02	3.01E+02	-2.414	0.01636 *			
	High Scales & Low Water Depth : EU Lineage	-1747.45	821.16	302.83	-2.128	0.03414 *			
	High Scales & High Water Depth : EU Lineage	-2306.73	824.55	300.96	-2.798	0.00548 **			
	Low Scales & High Water Depth : Gulf Lineage	-228.52	848.6	300.99	-0.269	0.78789			
	High Scales & Low Water Depth : Gulf Lineage	-1434.22	854.13	302.62	-1.679	0.09415 .			
	High Scales & High Water Depth : Gulf Lineage	389.86	858.15	300.88	0.454	0.64994			
Likelihood ratio test	intercept of null model	3713.48	444.65	50.02	8.352	4.76e-11 ***	36.24	11	0.0001544 ***
Rutin **Leaves Only** *selected by the literature*	intercept (Low Scales & Low Water, Delta Lineage)	6.29E+00	1.13E+00	1.58E+02	5.56E+00	1.11e-07 ***			
	Low Scales & High Water Depth	-2.25E+00	1.22E+00	3.63E+02	-1.84E+00	0.0663 .			
	High Scales & Low Water Depth	1.31E+00	1.19E+00	3.61E+02	1.105	0.2697			
	High Scales & High Water Depth	-2.72E+00	1.21E+00	3.61E+02	-2.248	0.0252 *			
	European lineage	-2.2393	1.1965	6.69E+01	-1.87E+00	0.0656 .			
	Gulf lineage	-2.2254	1.2227	71.1951	-1.82	0.0730 .			
	Replicates	4.79E-01	4.51E-01	3.63E+02	1.06E+00	0.2884			
	Low Scales & High Water Depth : EU Lineage	7.90E-01	1.66E+00	3.62E+02	0.477	0.6336			
	High Scales & Low Water Depth : EU Lineage	-0.6684	1.6387	361.6795	-0.408	0.6836			
	High Scales & High Water Depth : EU Lineage	1.2453	1.6465	360.8745	0.756	0.4499			
	Low Scales & High Water Depth : Gulf Lineage	2.4657	1.7426	361.7001	1.415	0.158			
	High Scales & Low Water Depth : Gulf Lineage	-0.6981	1.6878	360.7243	-0.414	0.6794			
	High Scales & High Water Depth : Gulf Lineage	1.5354	1.7028	360.5157	0.902	0.3678			
Likelihood ratio test	intercept of null model	4.2157	0.7981	124.8801	5.282	5.49e-07 ***	31.81	11	0.0008197 ***
Rutin **Roots Only** *selected by the literature*	intercept (Low Scales & Low Water, Delta Lineage)	2.51E+00	6.63E+00	1.76E+02	3.79E-01	0.705			
	Low Scales & High Water Depth	-7.30E-01	6.86E+00	3.05E+02	-1.06E-01	0.915			
	High Scales & Low Water Depth	8.35E-01	7.09E+00	3.01E+02	0.118	0.906			
	High Scales & High Water Depth	-5.33E-01	7.03E+00	3.05E+02	-0.076	0.94			
	European lineage	-0.43169	6.9205	9.01E+01	-6.20E-02	9.50E-01			
	Gulf lineage	-0.20286	7.20797	99.35322	-0.028	0.978			
	Replicates	-7.73E-01	2.56E+00	3.02E+02	-3.02E-01	0.762			
	Low Scales & High Water Depth : EU Lineage	3.62E-01	9.21E+00	3.04E+02	0.039	0.969			
	High Scales & Low Water Depth : EU Lineage	11.67152	9.3195	304.75363	1.252	0.211			
	High Scales & High Water Depth : EU Lineage	0.85842	9.4032	303.63293	0.091	0.927			
	Low Scales & High Water Depth : Gulf Lineage	-0.09847	9.67672	303.71357	-0.01	0.992			
	High Scales & Low Water Depth : Gulf Lineage	2.34009	9.7	304.93065	0.241	0.81			
	High Scales & High Water Depth : Gulf Lineage	0.01325	9.78833	303.46621	0.001	0.999			
Likelihood ratio test	intercept of null model	3.6791	4.0431	243.6012	0.91	0.364	8.51	11	6.70E-01
Trigonelline **Leaves Only** *selected by the literature*	intercept (Low Scales & Low Water, Delta Lineage)	8.75E+02	1.72E+02	3.66E+02	5.09E+00	5.63e-07 ***			
	Low Scales & High Water Depth	-1.50E+02	1.87E+02	3.66E+02	-8.00E-01	0.4241			
	High Scales & Low Water Depth	-3.58E+02	1.83E+02	3.66E+02	-1.96	0.0508 .			
	High Scales & High Water Depth	-2.01E+02	1.86E+02	3.66E+02	-1.083	0.2796			
	European lineage	-154.71	180.24	3.66E+02	-8.58E-01	3.91E-01			
	Gulf lineage	853.48	184.32	366	4.63	5.08e-06 ***			
	Replicates	-1.29E+02	6.93E+01	3.66E+02	-1.86E+00	0.0642 .			
	Low Scales & High Water Depth : EU Lineage	2.57E+02	2.55E+02	3.66E+02	1.01	0.3133			
	High Scales & Low Water Depth : EU Lineage	700.5	251.91	366	2.781	0.0057 **			
	High Scales & High Water Depth : EU Lineage	159.17	253.13	366	0.629	0.5299			
	Low Scales & High Water Depth : Gulf Lineage	270.15	267.88	366	1.008	0.3139			

Table S5 continued

	High Scales & Low Water Depth : Gulf Lineage	259.4	259.48	366	1	0.3181			
	High Scales & High Water Depth : Gulf Lineage	-425.61	261.79	366	-1.626	0.1049			
Likelihood ratio test	intercept of null model	1.03E+03	1.77E+02	2.07E+01	5.829	9.24e-06 ***	54.41	11	9.94e-08 ***
Trigonelline	intercept (Low Scales & Low Water, Delta Lineage)	8.97E+01	4.38E+01	6.94E+01	2.05E+00	0.0441 *			
**Roots Only**	Low Scales & High Water Depth	8.60E+01	4.17E+01	3.03E+02	2.06E+00	0.0398 *			
selected by	High Scales & Low Water Depth	-1.45E+01	4.33E+01	3.05E+02	-0.335	0.7381			
the literature	High Scales & High Water Depth	6.36E+01	4.26E+01	3.02E+02	1.492	0.1368			
	European lineage	-13.271	48.464	2.96E+01	-2.74E-01	7.86E-01			
	Gulf lineage	73.374	50.013	33.139	1.467	0.1518			
	Replicates	6.77E+00	1.54E+01	3.00E+02	4.38E-01	0.6617			
	Low Scales & High Water Depth : EU Lineage	-8.41E+00	5.58E+01	3.01E+02	-0.151	0.8803			
	High Scales & Low Water Depth : EU Lineage	16.44	56.653	303.411	0.29	0.7719			
	High Scales & High Water Depth : EU Lineage	-36.819	56.924	301.009	-0.647	0.5182			
	Low Scales & High Water Depth : Gulf Lineage	-35.865	58.584	301.048	-0.612	0.5409			
	High Scales & Low Water Depth : Gulf Lineage	-70.378	58.933	303.156	-1.194	0.2333			
	High Scales & High Water Depth : Gulf Lineage	70.881	59.246	300.89	1.196	0.2325			
Likelihood ratio test	intercept of null model	1.38E+02	2.96E+01	7.26E+01	4.644	1.49e-05 ***	51.48	11	3.382e-07 ***

Table S6. Summary statistics for metabolites targeted for herbivory. The estimate, standard error, *df*, *T*-value, and *P*-value columns represent the summary stats from the linear mixed model. The final three columns that include χ^2, *df*, and *P*-value represent the statistics from the ANOVA likelihood test that compared the null model to the linear mixed model.

response	predictors	Estimate	Std. Error	df	Tvalue	Pvalue	χ2	df	Pvalue
Jasmonic acid	intercept (Low Scales & Low Water, Delta Lineage)	2.27E+02	2.39E+01	6.82E+01	9.50E+00	4.08e-14 ***			
**Leaves Only**	Low Scales & High Water Depth	-1.17E+01	2.37E+01	3.61E+02	-4.94E-01	0.622			
selected by	High Scales & Low Water Depth	-1.18E+01	2.31E+01	3.61E+02	-0.509	0.611			
the literature	High Scales & High Water Depth	-1.90E+00	2.35E+01	3.61E+02	-0.081	0.936			
	European lineage	-24.374	26.738	2.91E+01	-9.12E-01	3.69E-01			
	Gulf lineage	129.602	27.196	30.93	4.766	4.22e-05 ***			
	Replicates	-1.01E+01	8.78E+00	3.62E+02	-1.15E+00	0.251			
	Low Scales & High Water Depth : EU Lineage	2.57E+01	3.22E+01	3.61E+02	0.799	0.425			
	High Scales & Low Water Depth : EU Lineage	-7.374	31.901	360.981	-0.231	0.817			
	High Scales & High Water Depth : EU Lineage	5.091	32.041	360.59	0.159	0.874			
	Low Scales & High Water Depth : Gulf Lineage	30.485	33.925	361.012	0.899	0.369			
	High Scales & Low Water Depth : Gulf Lineage	26.016	32.844	360.564	0.792	0.429			
	High Scales & High Water Depth : Gulf Lineage	-31.703	33.132	360.433	-0.957	0.339			
Likelihood ratio test	intercept of null model	260.569	28.316	13.34	9.202	3.81e-07 ***	33.71	11	0.000404 ***
Jasmonic acid	intercept (Low Scales & Low Water, Delta Lineage)	1.94E+02	3.41E+01	9.24E+01	5.70E+00	1.42e-07 ***			
**Roots Only**	Low Scales & High Water Depth	-7.21E+01	3.34E+01	3.03E+02	-2.16E+00	0.0317 *			
selected by	High Scales & Low Water Depth	-8.84E+01	3.47E+01	3.05E+02	-2.549	0.0113 *			
the literature	High Scales & High Water Depth	-8.10E+01	3.42E+01	3.03E+02	-2.368	0.0185 *			
	European lineage	-98.26	37.08	3.92E+01	-2.65E+00	0.0116 *			
	Gulf lineage	-41.23	38.38	44.02	-1.074	0.2886			
	Replicates	1.39E+01	1.24E+01	3.00E+02	1.12E+00	0.2639			
	Low Scales & High Water Depth : EU Lineage	6.68E+01	4.48E+01	3.02E+02	1.492	0.1368			
	High Scales & Low Water Depth : EU Lineage	116.17	45.44	304.19	2.556	0.0111 *			
	High Scales & High Water Depth : EU Lineage	79.48	45.7	301.63	1.739	0.0830 .			
	Low Scales & High Water Depth : Gulf Lineage	33.79	47.03	301.68	0.719	0.473			
	High Scales & Low Water Depth : Gulf Lineage	37.15	47.28	303.94	0.786	0.4326			
	High Scales & High Water Depth : Gulf Lineage	65.04	47.56	301.49	1.367	0.1725			
Likelihood ratio test	intercept of null model	118.31	20.53	173.32	5.762	3.73e-08 ***	14.98	11	1.84E-01
Methyl jasmonate	intercept (Low Scales & Low Water, Delta Lineage)	3.07E+02	7.07E+01	5.92E+01	4.35E+00	5.45e-05 ***			
**Leaves Only**	Low Scales & High Water Depth	1.20E+02	6.94E+01	3.61E+02	1.73E+00	0.0848 .			
selected by	High Scales & Low Water Depth	9.68E+01	6.76E+01	3.60E+02	1.432	0.153			
the literature	High Scales & High Water Depth	6.51E+01	6.87E+01	3.60E+02	0.948	0.3437			
	European lineage	90.32	79.66	2.56E+01	1.13E+00	2.67E-01			
	Gulf lineage	-104.25	80.98	27.17	-1.287	0.2088			
	Replicates	1.62E+01	2.57E+01	3.62E+02	6.30E-01	0.5294			
	Low Scales & High Water Depth : EU Lineage	-1.16E+02	9.42E+01	3.61E+02	-1.234	0.218			
	High Scales & Low Water Depth : EU Lineage	-129.49	93.21	360.68	-1.389	0.1656			
	High Scales & High Water Depth : EU Lineage	-101.6	93.62	360.3	-1.085	0.2785			
	Low Scales & High Water Depth : Gulf Lineage	-116.47	99.13	360.71	-1.175	0.2408			
	High Scales & Low Water Depth : Gulf Lineage	-168.28	95.97	360.28	-1.754	0.0804 .			
	High Scales & High Water Depth : Gulf Lineage	-22.14	96.81	360.15	-0.229	0.8192			
Likelihood ratio test	intercept of null model	321.28	53.18	37.58	6.042	5.2e-07 ***	18.21	11	0.07674 .

Table S6 continued

Methyl jasmonate	intercept (Low Scales & Low Water, Delta Lineage)	1.34E+01	1.68E+00	5.17E+01	7.98E+00	1.42e-10 ***			
**Roots Only**	Low Scales & High Water Depth	-3.68E+00	1.53E+00	3.02E+02	-2.41E+00	0.01674 *			
selected by	High Scales & Low Water Depth	-3.04E+00	1.59E+00	3.04E+02	-1.911	0.05690 .			
the literature	High Scales & High Water Depth	-2.34E+00	1.56E+00	3.02E+02	-1.499	0.13495			
	European lineage	-4.6019	1.9142	2.34E+01	-2.40E+00	0.02450 *			
	Gulf lineage	-4.845	1.9673	25.9357	-2.463	0.02075 *			
	Replicates	6.35E-01	5.66E-01	3.00E+02	1.12E+00	0.26328			
	Low Scales & High Water Depth : EU Lineage	2.67E+00	2.05E+00	3.01E+02	1.303	0.1936			
	High Scales & Low Water Depth : EU Lineage	5.6249	2.0794	302.6312	2.705	0.00722 **			
	High Scales & High Water Depth : EU Lineage	2.0571	2.0878	300.8019	0.985	0.32527			
	Low Scales & High Water Depth : Gulf Lineage	1.0362	2.1487	300.8254	0.482	0.62997			
	High Scales & Low Water Depth : Gulf Lineage	2.1158	2.1629	302.4222	0.978	0.32873			
	High Scales & High Water Depth : Gulf Lineage	1.3601	2.1728	300.7178	0.626	0.5318			
Likelihood ratio test	intercept of null model	9.2303	1.1016	50.5	8.379	4.02e-11 ***	24.65	11	0.01025 *
Methyl	intercept (Low Scales & Low Water, Delta Lineage)	3.68E+02	6.15E+01	2.24E+01	5.99E+00	4.66e-06 ***			
dihydrojasmonate	Low Scales & High Water Depth	3.90E+01	4.99E+01	3.61E+02	7.81E-01	0.4355			
**Leaves Only**	High Scales & Low Water Depth	6.60E+01	4.86E+01	3.60E+02	1.357	0.1755			
selected by	High Scales & High Water Depth	9.96E+00	4.94E+01	3.60E+02	0.202	0.8404			
the literature	European lineage	162.928	75.332	1.28E+01	2.16E+00	0.0501 .			
	Gulf lineage	-114.059	76.067	13.283	-1.499	0.1571			
	Replicates	-7.59E+00	1.85E+01	3.61E+02	-4.11E-01	0.6814			
	Low Scales & High Water Depth : EU Lineage	-9.16E+01	6.78E+01	3.61E+02	-1.352	0.1772			
	High Scales & Low Water Depth : EU Lineage	-121.491	67.065	360.505	-1.812	0.0709 .			
	High Scales & High Water Depth : EU Lineage	-61.648	67.347	360.359	-0.915	0.3606			
	Low Scales & High Water Depth : Gulf Lineage	27.757	71.32	360.521	0.389	0.6974			
	High Scales & Low Water Depth : Gulf Lineage	-98.204	69.034	360.36	-1.423	0.1557			
	High Scales & High Water Depth : Gulf Lineage	57.736	69.635	360.303	0.829	0.4076			
Likelihood ratio test	intercept of null model	388.8	47.31	19.46	8.218	9.41e-08 ***	19.99	11	0.04546 *
Methyl	intercept (Low Scales & Low Water, Delta Lineage)	4.82E+01	1.85E+01	7.73E+01	2.61E+00	0.0109 *			
dihydrojasmonate	Low Scales & High Water Depth	-2.31E+01	1.78E+01	3.03E+02	-1.30E+00	0.1955			
**Roots Only**	High Scales & Low Water Depth	6.28E+00	1.85E+01	3.05E+02	0.34	0.7344			
selected by	High Scales & High Water Depth	-1.58E+01	1.82E+01	3.03E+02	-0.868	0.3862			
the literature	European lineage	0.1391	20.3186	3.27E+01	7.00E-03	9.95E-01			
	Gulf lineage	-10.3661	20.9935	36.6604	-0.494	0.6244			
	Replicates	2.09E+00	6.61E+00	3.00E+02	3.16E-01	0.7524			
	Low Scales & High Water Depth : EU Lineage	-1.66E+00	2.39E+01	3.02E+02	-0.069	0.9448			
	High Scales & Low Water Depth : EU Lineage	68.0738	24.2376	303.7187	2.809	0.0053 **			
	High Scales & High Water Depth : EU Lineage	11.5247	24.361	301.1949	0.473	0.6365			
	Low Scales & High Water Depth : Gulf Lineage	19.5157	25.0714	301.2396	0.778	0.4369			
	High Scales & Low Water Depth : Gulf Lineage	25.3626	25.2139	303.4615	1.006	0.3153			
	High Scales & High Water Depth : Gulf Lineage	18.231	25.3548	301.0656	0.719	0.4727			
Likelihood ratio test	intercept of null model	49.071	11.935	132.94	4.111	6.85e-05 ***	57.27	11	2.973e-08 ***
